# Neural Correlates of Goal‐Directed Preparation to Switching Across External and Internal Domains

**DOI:** 10.1002/hbm.70376

**Published:** 2025-10-15

**Authors:** Sara Calzolari, Brandon T. Ingram, Andrew P. Bagshaw, Davinia Fernández‐Espejo

**Affiliations:** ^1^ Centre for Human Brain Health University of Birmingham Birmingham UK; ^2^ School of Psychology University of Birmingham Birmingham UK

**Keywords:** DCM, DMN, fMRI, frontoparietal, self‐referential processing, task‐switching

## Abstract

While it is well accepted that the human brain shifts between internal and external monitoring both during tasks and at rest, no task‐switching studies have focused on brain changes when switching from and to self‐referential processing. Using a cued task‐switching design, we explored the preparatory fMRI activation associated with switching not only within externally oriented tasks, but also within self‐referential tasks, as well as between these two domains. We found that preparing to perform internal tasks activated the default mode network, while preparing for external tasks activated regions of the dorsal attention network (DAN). Switch preparation activated left‐lateralised DAN regions with ventrolateral peaks as well as dorsal precuneus, posterior cingulate and supplementary motor area. These results show a dynamic pattern of communication across networks associated with external and internal domain processing and common preparatory activation in working memory and executive control regions. In particular, the dorsal precuneus was consistently engaged in task‐switch preparation, suggesting a key role of this region in cognitive control, in the context of switching across external and internal domains.

## Graphical abstract

We used a cued task switching fMRI study to investigate switches between internally and externally oriented tasks. Switch preparation was associated with activation of frontoparietal areas (especially dorsal precuneus), including regions belonging to the default mode and dorsal attention networks.

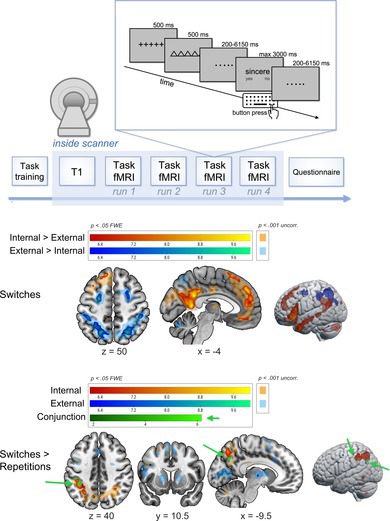

## 1 Introduction

Our brain constantly encodes and coordinates information coming from the external environment with internal signals from our body and inner thoughts. Seminal research identified distinct functional brain networks underpinning external and internal processing (Fox and Raichle 2007). Specifically, the default mode network (DMN) is associated with self‐related thoughts, spontaneous cognition and mind‐wandering, autobiographical memory and simulation of events (Andrews‐Hanna 2012; Buckner et al. 2008; Raichle 2015). In contrast, the dorsal attention network (DAN) is associated with externally oriented attention and working memory (Corbetta and Shulman 2002; Miller and Buschman 2013). These networks are traditionally thought to be anti‐correlated, as they show opposite patterns of activation during rest (Vanhaudenhuyse et al. 2010) as well as attentional (Weissman et al. 2006) and self‐referential tasks (Davey et al. 2016; Kelley et al. 2002; Whitfield‐Gabrieli et al. 2011). However, on occasions, the DMN is also involved in task preparation (Hampshire et al. 2019; Koshino et al. 2014) and demanding cognitive shifts between tasks (Crittenden et al. 2015), suggesting a DMN contribution to goal‐directed cognition as well. Recent accounts, in fact, highlight the variable nature of DMN–DAN interactions as a function of the cognitive context (Dixon et al. 2017).

The relationship between DMN and DAN is thought to be coordinated by executive control and/or salience networks (ECN and SN, sometimes collectively referred to as the frontoparietal control network) (Gao and Lin 2012; Menon and Uddin 2010; Seeley et al. 2007; Spreng et al. 2010). In task‐switching paradigms, commonly used to investigate neural dynamics underlying goal‐directed shifts, preparing to switch to a different task indeed elicits activation in a set of lateral frontoparietal regions generally overlapping with the ECN (i.e., inferior frontal gyrus, inferior frontal junction [IFJ], pre‐supplementary motor area [SMA], intraparietal sulcus and posterior parietal cortex), along with task‐specific areas (e.g., fusiform gyrus for colour encoding) (Brass and von Cramon 2004; Wylie et al. 2006). In addition, animal studies revealed a fundamental contribution of the mediodorsal (MD) thalamus (which displays wide functional connections with all the above‐mentioned networks) in supporting and boosting cortical representations during cognitive switching (Rikhye et al. 2018; Schmitt et al. 2017). The role of the MD thalamus has yet to be confirmed in neuroimaging studies in humans.

Crucially, these studies only included externally oriented tasks (e.g., parity and magnitude judgement tasks, letter identification, colour or object naming, etc.; Kiesel et al. 2010) and, to our knowledge, no research has previously focused on understanding how these networks interact to allow goal‐directed cognitive shifts across external and internal (self‐referential) processes.

In an earlier study, we developed a cued task‐switching paradigm (Calzolari et al. 2022) to characterise behavioural switches between external and internal processing. We provided the first evidence of switch costs (higher RTs for switches compared to repetitions) across internal tasks and of additional costs associated with between‐domain (i.e., from internal to external and vice versa) compared to within‐domain switches (Calzolari et al. 2022). This suggested the presence of two domain‐specific control subsystems that communicate and share common features of cognitive control (possibly via a higher‐order control system).

Here, we built upon our behavioural findings to address the neural correlates of task‐switch preparation when both externally and internally oriented tasks are involved using fMRI. We aimed to replicate our earlier behavioural results and hypothesised (Hypothesis 1) that we would identify DMN and DAN activation in preparation for internal and external tasks respectively. We also hypothesised (Hypothesis 2) activation in areas associated with cognitive control and working memory (i.e., ECN/DAN regions) in preparation for switches compared to task repetitions and (Hypothesis 3) to see common neural activations associated with switching in both domains (i.e., an overarching control system), including the MD thalamus. We also explored whether our hypothesised activation patterns would differ when looking at between‐domain and within‐domain switches, expecting differential activation between switch types (thus mirroring the behavioural results from our previous study) possibly located in domain‐general control areas (Hypothesis 4). Finally, we explored modulations in effective connectivity in response to different types of switches to shed light on the causal directed relationships between regions during network reorganisation.

## 2 Methods

### 2.1 Participants

We recruited a total of 33 participants (mean age = 22.61 ± 2.83 years, range 18–30 years; 22 women, 11 men) via the University of Birmingham Research Participation Scheme and advertisements across campus. Participants were right‐handed, native English speakers and reported no history of neurological or psychiatric conditions. They also met the eligibility criteria to enter the MRI environment. Participants had the chance to read the information sheet and ask questions before starting the experiment. They filled in the MRI screening form and gave written informed consent before MRI scanning. They received compensation in the form of cash or research credits. The University of Birmingham's Science, Technology, Engineering and Mathematics Ethical Review Committee provided approval for our study.

We discarded data from two participants due to low accuracy (< 60% of correct responses) in one or more task conditions and 1 participant due to technical issues in the post‐scan questionnaire. This resulted in a total of 30 participants (mean age = 21.50 ± 2.90 years; 21 women, 9 men) included in the analysis.

### 2.2 Experimental Design and Procedure

**FIGURE 1:**
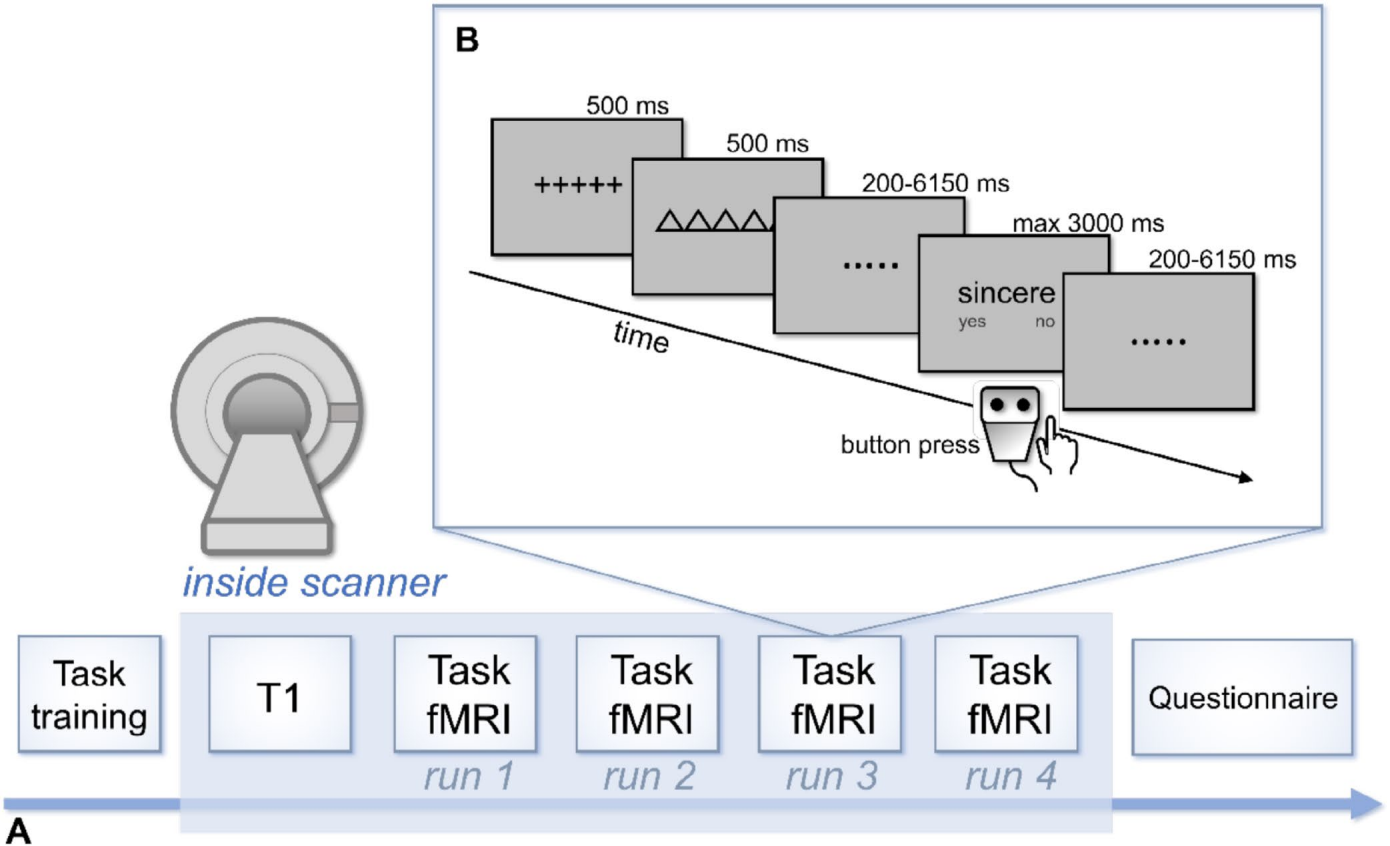
Experimental design. (A) Structure of the main experimental session including an initial task training phase outside the MRI scanner, a structural T1 scan, and four runs of task‐fMRI. A post‐task questionnaire immediately after scanning allowed us to obtain accuracy for the internally oriented tasks. (B) Structure of a single trial: after a 500 ms fixation screen, a geometrical cue (500 ms) signalled participants which of the four tasks to perform on the following word stimulus. After a jittered interval (pseudologarithmic jittering), a written adjective appeared and stayed at the centre of the screen until button press, for a maximum of 3 s. Participants had to respond yes or no using a button box with their right hand. After response, another jittered interval separated the trial from the following one.

The main experiment consisted of one session (Figure 1A) lasting approximately 2.5 h. We provided participants with both written and oral task instructions and gave them the chance to ask questions. They then performed an initial training session of 10 min outside of the scanner. Once inside the scanner, they first underwent a T1‐weighted structural scan (approximately 7 min), then performed the main task (approximately 50 min divided into 4 runs, see the next subsection for details). After the scanning session, participants completed a questionnaire (10 min) aimed at verifying the correct responses for the two internal task conditions to use for further analyses. This instructed them to perform the ‘personality’ and ‘current sensations’ judgements again but in a self‐paced way and using a list that displayed all stimuli. For the ‘current sensations’ judgements, participants were instructed to think about how they felt when they were inside the scanner.

### 2.3 Task and Stimuli

We used the same task as in our previous study (Calzolari et al. 2022), but presented in the MRI scanner via custom MATLAB scripts (version R2018b) and Psychtoolbox‐3 (Kleiner et al. 2007). In brief, the experiment was a cued task‐switching paradigm with written English words (personality trait and bodily adjectives) as stimuli. Based on a geometric cue appearing in advance (with pseudologarithmically jittered intervals), participants had to either direct their attention towards specific stimulus features in ‘external’ tasks (i.e., to assess if the third letter of the stimulus was a consonant or if the penultimate letter was a vowel) or they needed to access self‐related characteristics in ‘internal’ tasks (i.e., judge if the stimulus described their own personality, or if it described a present bodily sensation). Participants responded using the index and middle fingers of their right hand and by pressing either the left (for ‘yes’) or right (for ‘no’) buttons of a NAtA Technologies Inc. response box. The jittered cue‐to‐target and inter‐trial intervals allowed for an optimal separation between cue‐related and target‐related activity, as well as between trials activity (De Baene and Brass 2011).

The main experimental phase inside the scanner (380 trials) was divided into four runs (95 trials each, lasting 13 min and 12 s). Participants saw the task instructions at the beginning of each run and had the chance to rest for a few minutes between runs. A full description of the task paradigm, parameters and stimuli can be found in our previous study (Calzolari et al. 2022). Figure 1B offers a representation of the trial structure.

### 2.4 Behavioural Analysis

We analysed the behavioural data using JASP 0.16.3 (JASP Team 2020), with the same analytical steps we used in our previous study (Calzolari et al. 2022). We did not discard any participants due to missing trials (no one missed 10% of trials or more; mean percentage of missing trials = 0.49% ± 0.80%), but discarded two participants with less than 60% accuracy in any of the four tasks. We removed outlier trials (Tukey's method; Tukey 1977) and trials with RTs below 200 ms, resulting in an average of 3.44% (± 1.33) discarded trials per participant, in addition to the trials discarded due to incorrect responses (7.30% ± 4.22%).

We performed two main analyses: a 2 × 2 rANOVA (frequentist and Bayesian) with domain (internal, external) and trial type (repetitions, switches) as factors on mean RTs and a 2 × 2 rANOVA with factors domain (internal, external) and switch type (within‐domain, between‐domains) on switch costs. We also performed one‐sample *t* tests comparing each type of switch costs in this last ANOVA to 0. We included accurate trials only (i.e., correct responses) and excluded the data from the training phase (outside the MRI). We obtained accuracy for the two internally oriented tasks by comparing responses during the main experiment with responses to the post‐task questionnaire. Responses were considered correct if they matched the answers given in the post‐task questionnaire.

### 2.5 MRI Acquisition

We acquired the data on a Siemens MAGNETOM Prisma 3T system at the Centre for Human Brain Health (CHBH, University of Birmingham) using a 64‐channel head coil.

We collected the task fMRI data in 4 separate runs, all with a 2D GRE single‐shot SMS‐EPI sequence and the following parameters: 528 volumes per run, 57 slices, TR = 1500 ms, TE = 35 ms, voxel size = 2.5 × 2.5 × 2.5 mm, flip angle = 71°, matrix size = 84 × 84 × 57, field of view = 210 × 210 mm, iPAT acceleration factor = 3. We also obtained a T1‐weighted 3D MPRAGE image for anatomical co‐registration and spatial normalisation with parameters TR = 2400 ms, TE = 2.22 ms, flip angle = 8°, matrix size = 208 × 300 × 320, field of view = 256 × 256, voxel size = 0.8 × 0.8 × 0.8 mm.

### 2.6 fMRI Preprocessing

**FIGURE 2:**
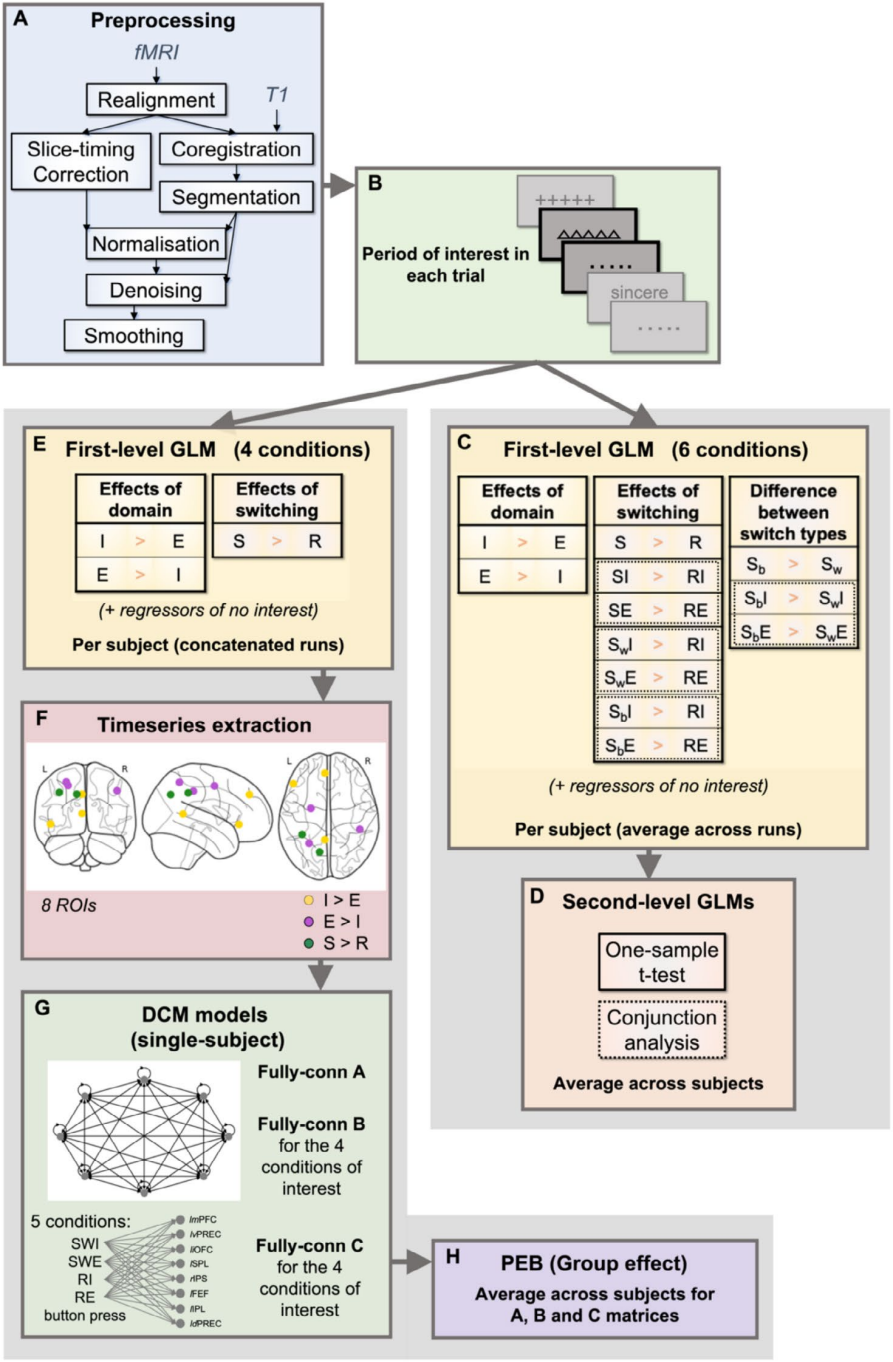
fMRI preprocessing and analysis pipeline. (A) A standard preprocessing pipeline was implemented on SPM12, with the addition of a denoising step (TAPAS PhysIO toolbox) which modelled physiological noise such as cardiac and respiratory activity. This was regressed out in the first‐level GLM along with movement regressors. (B) The analysis focused on the cue‐to‐target interval (correct trials only) to capture the anticipatory activation in preparation for task performance. (C) The tables show the pairwise contrasts performed in the first‐level GLMs (per subject, averaging across runs): E = all trials with external tasks; I = all trials with internal tasks; R = all repetitions; RE = repetition of external tasks; RI = repetition of internal tasks; S = all switches; S_b_E = switches from internal to external tasks (between‐domain); S_b_I = switches from external to internal tasks (between‐domain); SE = all switches towards external tasks; SI = all switches towards internal tasks; S_w_E = switches within external tasks; S_w_I = switches within internal tasks. (D) A one‐sample *t* test at the second level then computed the group average effect of each contrast. We also performed conjunction analyses (contrasts marked with a dotted line) to highlight patterns of activation common to both the internal and external domains. (E) Tables show the pairwise contrasts performed in the new GLM (four conditions only: Internal repetitions, external repetitions, switches to internal, switches to external) which were used to concatenate the runs within each subject and obtain activation maps for timeseries extraction. (F) Timeseries extraction from 8 ROIs. (G) Individual DCM models including four conditions + button press, fully connected A, B and C matrices (B and C matrices were specified for each of the four conditions of interest). Abbreviations: *ld*PREC = left dorsal precuneus; *l*FEF = left frontal eye fields; *li*OFC = left inferior orbitofrontal cortex; *l*IPL = left inferior parietal lobule; *lm*PFC = left medial prefrontal cortex; *l*SPL = left superior parietal lobule; *lv*PREC = left ventral precuneus; rIPS = right intraparietal sulcus. (H) Individual models were averaged across participants under the Parametric Empirical Bayes framework to obtain effective connectivity at the group level.

We preprocessed and analysed the fMRI data using SPM12 version v7771 (Friston et al. 2007) on MATLAB R2019b. The pipeline (Figure 2) followed standard preprocessing steps (realignment, slice timing correction, coregistration between anatomical and functional images, spatial normalisation and smoothing with an 5 mm^3^ Gaussian kernel), plus the modelling and removal of physiological noise using the TAPAS PhysIO Toolbox (Kasper et al. 2017), a denoising procedure that resembles the aCompCor method (Behzadi et al. 2007). Specifically, the procedure involved a principal component analysis (PCA) on the segmented white matter and cerebrospinal fluid (CSF) masks, based on the assumption that the temporal fluctuations in those regions are related to respiratory and cardiac artifacts (Behzadi et al. 2007). White matter and CSF ROIs were extracted using a 99% and 85% threshold for voxel inclusion, respectively, and cropping one voxel (mask erosion). We computed the first five PCs (default setting in the toolbox) and added them as noise regressors (along with movement) in our subsequent general linear model (GLM). Within the PhysIO toolbox, we also performed motion censoring on volumes with more than 2 mm translation and/or 2° rotation.

### 2.7 GLM Analysis

We based the fMRI analysis on the six task conditions of interest in this design: repetitions of internal tasks, repetitions of external tasks, switches within internal tasks, switches within external tasks, switches from external to internal (i.e., between‐domain switches towards internal tasks) and from internal to external (i.e., between‐domain switches towards external tasks). Importantly, for these analyses, we focused on the cue‐to‐target period only (700–6650 ms) to investigate preparatory activation and not task performance itself. We only included correct trials within these analyses.

We conducted a whole‐brain analysis using the general linear statistical model (GLM; Friston et al. 1994) on SPM12. At the first level (single subject), the GLM model included the six task conditions mentioned above as regressors of interest.

All main pairwise contrasts are provided in Table S1.

Contrasts 1 and 2 assessed the difference between domains (internal vs. external) regardless of trial type (switches/repetitions). Contrast 3 investigated the activity in preparation for any switch as compared to repetitions, while Contrasts 4 and 5 looked at domain‐specific effects on switching. In addition, we performed more nuanced comparisons to explore the effect of each specific switch type as compared to repetitions (Contrasts 6–9). Finally, Contrasts 10–12 explored the differences across switch types. All contrasts were replicated across runs so that the effects were averaged across the four experimental blocks. All GLMs included a regressor of no interest with the onsets of the button presses (duration of zero) to regress out the motor activity associated with responding, as well as the realignment parameters, censored volumes and physiological noise regressors from the PhysIO toolbox as effects of non‐interest. We used the default SPM high‐pass filter with a cut‐off period of 128 s to remove slow‐signal drifts. We then performed second‐level GLMs with a one‐sample *t* test design in order to test the group effects for each of our contrasts of interest. In addition, we performed a conjunction analysis for each of the four pairs of first‐level contrasts. We carried out these conjunction analyses in separate second‐level GLMs by selecting a one‐way ANOVA design with two groups (one for each first‐level pairwise contrast) and contrasts that separately computed the average of each group. These contrasts were then selected for conjunction analysis in SPM, for which we chose the conjunction null option to only retain the activation common to both contrasts (Nichols et al. 2005).

We report voxels that survived family‐wise error (FWE) correction with *p* < 0.05 at the voxel level as statistically significant. We do not report clusters with two significant voxels or less.

### 2.8 DCM Analysis

#### 2.8.1 Region Selection and Timeseries Extraction

First, we performed a first‐level GLM where we concatenated the four runs for each subject. Given the results from the main GLM (see Section 3), we decided to collapse across within‐ and between‐domain switches to obtain a simpler design that included four conditions (internal repetitions, external repetitions, all switches to internal, all switches to external) and simplify our model space. We then extracted eight regions of interest (ROI) corresponding to the main activation clusters from the main GLM. In particular, since our goal was to investigate the connectivity between DMN and DAN and potentially spot the influence of domain‐general control regions, we used the T‐contrast ‘internal > external’ to extract left‐lateralised mPFC, ventral precuneus and inferior orbitofrontal cortex (OFC), while we used the *T*‐contrast ‘external > internal’ to extract left superior parietal, right supramarginal and left superior frontal cortex (frontal eye fields, FEF). We also used the *T*‐contrast ‘switches > repetitions’ to extract the activity of left IPL and dorsal precuneus regions. Since we could not find thalamic activation in these GLM contrasts, the MD thalamus was not included as an ROI despite our original hypothesis. To derive the ROI coordinates, we identified the cluster peaks in the activation maps obtained from the above‐mentioned contrasts in the previous GLM. These group coordinates served as a starting point to search for the individual peaks in the activation maps of the new GLM. Individual peaks were constrained to be within a 10 mm radius (twice the FWHM smoothing kernel) from the group coordinate and exceed the statistical threshold of *p* < 0.001 uncorrected. When no peak was found with this threshold, we reduced it by 0.05 until a minimum threshold of 0.15 was reached, as suggested by (Zeidman, Jafarian, Corbin, et al. 2019) and done previously in (Aloi et al. 2022; Calzolari et al. 2023). We then extracted the timeseries in a sphere of 6 mm radius from the individual coordinates. We adjusted the timeseries using an *F*‐contrast that identified the effects of interest (i.e., the four task conditions), regressing out any other nuisance factor, that is, head motion, physiological noise, button presses.

#### 2.8.2 Individual Level DCM Specification and Estimation

For each participant, we specified an individual bilinear, one‐state, deterministic DCM model. We included the eight ROIs and specified four conditions of interest (internal repetitions, external repetitions, all switches to internal, all switches to external), mean‐centering the inputs. We specified a fully connected model where all extrinsic and intrinsic connectivity parameters were switched on (‘matrix A’) and we set each condition as driving input to all regions (‘matrix C’). Each condition was also set as modulatory input on the entire fully connected network (‘matrix B’). The choice of specifying a fully connected B matrix is not standard (commonly, the task is set to modulate self‐connections only) but is justified by the nature of our paradigm and research questions. Specifically, as it allowed us to investigate whether the preparation of switches towards the internal or the external domain can modulate the effective connectivity from domain‐general regions towards domain‐specific regions. We also included button presses in the model, which were, however, not set as either driving nor modulatory inputs. This model yielded the most positive free energy among the models we designed (strong Bayesian evidence given by a difference in free energy greater than 3). See (Friston et al. 2023) for more information on the free energy principle.

#### 2.8.3 Group Level Parametrical Empirical Bayes

Within the PEB framework, we computed the average of individual DCM models across participants and performed an automatic search across reduced models (Zeidman, Jafarian, Seghier, et al. 2019). We then filtered the resulting parameters by retaining those that exceed 95% of posterior probability (equivalent to a Bayes factor of 3 and considered strong evidence).

See Figure 2 for a summary of the analysis pipeline, including preprocessing, GLM and DCM procedures.

## 3 Results

### 3.1 Behavioural Results

Participants performed the experiment with high accuracy (overall mean score of 92.90% ± 4.47%). The mean accuracy for the external tasks (consonant and vowel finding) was 94.90% (± 4.71) and 96.77% (± 2.98), respectively, while internal tasks (personality and current sensations) resulted in 91.35% (± 8.61) and 88.74% (± 7.23) mean accuracy, respectively.

**FIGURE 3:**
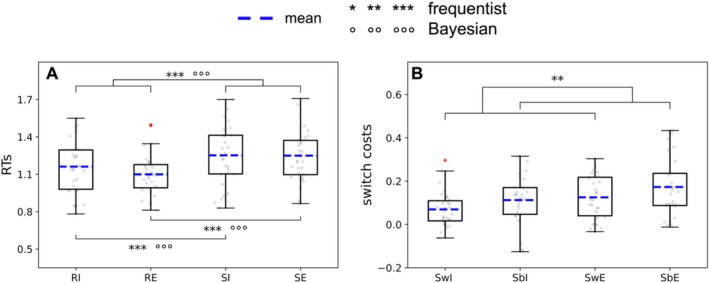
Boxplots showing the results from the analyses on RTs. (A) main effect of trial type and post hoc *t* tests from the 2 × 2 rANOVA with factors domain (internal, external) and trial type (switches, repetitions); (B) main effect of switch type from the 2 × 2 rANOVA on switch costs with factors domain and switch type (within, between domain). Outlier participants are represented as red dots, but were not excluded from the analyses. The mean is represented as a blue dashed line. The whiskers represent the range between minimum and maximum, that is, the variability outside the upper and lower quartiles. *p < 0.05; **p < 0.01, ***p < 0.001, °BF10 > 3 (substantial evidence), °°BF10 > 10 (strong evidence), °°°BF10 > (very strong evidence). Abbreviations: RI = repetition of internal tasks; RE = repetition of external tasks; SI = all switches towards internal tasks; SE = all switches towards external tasks; S_w_I = switches within internal tasks; S_w_E = switches within external tasks; S_b_I = switches from external to internal tasks (between‐domain); S_b_E = switches from internal to external tasks (between‐domain).

The first 2 × 2 rANOVA on RTs (factors domain and trial type; Figure 3A) resulted in a significant main effect of trial type (BF_10_ = 5.581e + 7, *F*(1,29) = 108.21, *p* < 0.001, $\eta_{p}^{2}$ = 0.79) whereby switches were significantly slower than repetitions, in line with our previous behavioural study (Calzolari et al. 2022). There was no main effect of domain (BF_10_ = 0.56, *F*(1,29) = 0.85, *p* = 0.36, $\eta_{p}^{2}$ = 0.029). The interaction between domain and trial type was significant, again in line with our previous results (BF_10_ = 7.945, *F*(1,29) = 7.57, *p* = 0.01, $\eta_{p}^{2}$ = 0.21). Post hoc *t* tests confirmed the presence of switch costs in both the external (BF_10_ = 1.195e+7, *t* = 9.47, *p*_bonf_ < 0.001) and the internal domain (BF_10_ = 16658.084, *t* = 5.70, *p*_bonf_ < 0.001).

The 2 × 2 rANOVA on switch costs (factors domain and switch type; Figure 3B) resulted in a significant main effect of domain (greater costs for switches to external than to internal tasks; BF_10_ = 4.02, *F*(1,29) = 7.09, *p* = 0.013, $\eta_{p}^{2}$ = 0.20) and switch type (BF_10_ = 2.92, *F*(1,29) = 9.44, *p* = 0.005, $\eta_{p}^{2}$ = 0.25). There was no significant interaction between domain and switch type (BF_10_ = 0.33, *F*(1,29) = 0.02, *p* = 0.894, $\eta_{p}^{2}$ = 6.217e−4). The main effect of switch type indicates additional costs when switching domains compared to staying within domain. However, post hoc *t* tests did not show such additional costs in neither of the two domains: switching from external to internal domain was not slower than staying within the internal domain, although the Bayesian *t* test showed anecdotal evidence in favour of a difference (BF_10_ = 2.07, *t* = 1.83, *p*_bonf_ = 0.434) and switching from internal to external domain was not slower than staying within the external domain (BF_10_ = 0.73, *t* = 2.04, *p*_bonf_ = 0.276). This was against our predictions as well as our previous behavioural study (Calzolari et al. 2022). Nonetheless, it is important to notice that all four switch types were significantly different from repetitions (i.e., different from 0 in the one‐sample *t* tests: all *p* < 0.001), including the switches within the internal domain (BF_10_ = 396.80, *t* = 4.67, *p* < 0.001, *d* = 0.85) and the between‐domain switches from external to internal tasks (BF_10_ = 6035.42, *t* = 5.75, *p* < 0.001, *d* = 1.05) (see Table S2).

Table 1 displays the mean and standard deviation of all conditions (RTs after subtraction of task repetitions are reported, where relevant).

**TABLE 1:** Mean and SD of RTs for all conditions.

	Repetitions (s)	Combined switches (s)	Within domain difference (s)	Between domains difference (s)
All	1.129 (0.165)	1.251 (0.197)		
Internal	1.161 (0.220)	1.252 (0.235)	0.070 (0.081)	0.112 (0.107)
External	1.099 (0.168)	1.250 (0.205)	0.125 (0.099)	0.173 (0.137)

*Note:* In the case of within‐ and between‐domain switches, we report the mean difference between their RTs and those of task repetitions, since that is what we used to perform that analysis. Raw RTs for those conditions can be found in Table S3. All units are seconds.

### 3.2 fMRI Results

Here we describe all the statistically significant clusters (FWE‐corrected *p* < 0.05) with a size of at least 10 voxels. Full results including smaller clusters (minimum three voxels) are reported in the tables. The anatomical labels corresponding to peak activation were taken from the third version of the automated anatomical labelling atlas (AAL3; Rolls et al. 2020).

**FIGURE 4:**
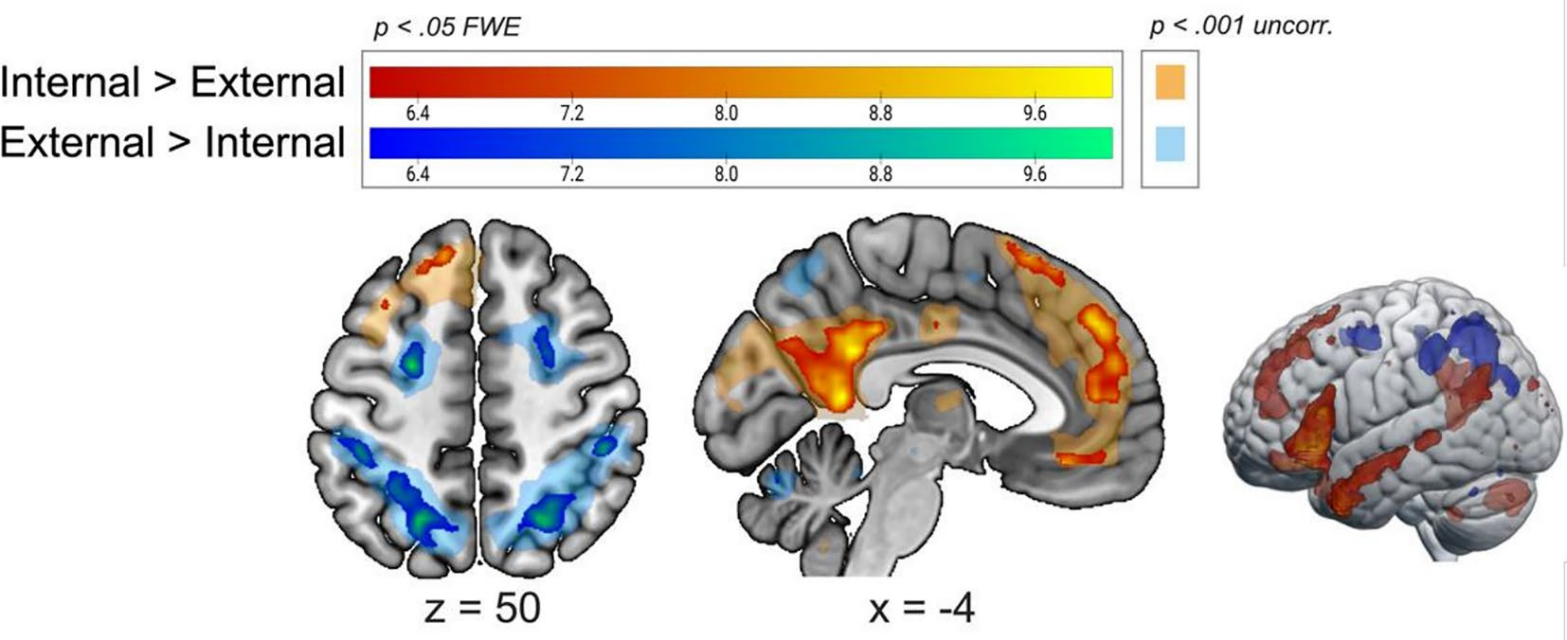
Difference in brain activation between domains. Activation at the group level (one‐sample *t* test) for the whole‐brain comparisons between domains, regardless of trial type. Specifically, the first‐level GLMs compared ‘internal > external’ and ‘external > internal’ on a multi‐run model for each subject (four runs of task per participant). The activation maps are shown at *p* < 0.05 FWE‐corrected (bright colours; warm scale: Internal > external; cold scale: external > internal) and rendered on a standard template (mni152 template in MRIcroGL). Uncorrected activation maps at *p* < 0.001 are also shown for representational purposes with faint, transparent colours. For each slice, the relative MNI coordinate is reported.

Starting from the effects of domain (Figure 4), comparing the preparation of all internal task trials versus all external task trials revealed activation mostly in DMN regions (various portions of left medial prefrontal cortex, precuneus, angular gyrus, bilateral middle temporal gyrus), as well as left inferior OFC and right cerebellum. See Table 2A for a full list of results. The opposite comparison (external > internal > trials) instead resulted in the activation of DAN regions such as bilateral superior frontal lobes (FEF), left superior and inferior parietal lobules (SPL and IPL), right angular and supramarginal gyri (Table 2B). These results are in line with our Hypothesis 1 (see Figure S1 and Table S4) for the results of these contrasts separately for repetition and switch trials.

**TABLE 2:** fMRI results on the effects of domain.

Contrast	Region (AAL3)	Peak coordinates	Voxels per cluster	*z*‐score	Peak *p*_FWE‐corr_	Cluster p_FWE‐corr_
I > E	L inferior orbitofrontal	−49.5, 30.5, −5	563	7.066	< 0.001	0.000
	R crus I	30.5, −84.5, −32.5	396	6.835	< 0.001	0.000
	L precuneus	−4.5, −49.5, 10	427	6.675	< 0.001	0.000
	L rectus (medial frontal)	−2, 33, −17.5	87	6.606	< 0.001	< 0.001
	L medial superior frontal	−4.5, 45.5, 37.5	505	6.503	< 0.001	0.000
	L middle temporal	−57, −34.5, 0	73	6.457	< 0.001	< 0.001
	L angular	−49.5, −57, 25	167	6.410	< 0.001	0.000
	R middle temporal pole	45.5, 18, −27.5	42	6.340	< 0.001	
	L inferior temporal	−47, 5.5, −35	370	6.247	< 0.001	0.000
	R cerebellar tonsil	5.5, −52, −45	17	6.224	< 0.001	< 0.001
	R middle temporal	53, −9.5, −17.5	19	5.787	< 0.001	< 0.001
	L mid frontal	−34.5, 18, 52.5	4	5.652	< 0.001	0.001
	L inf frontal, pars triangularis	53, 28, 12.5	18	5.445	0.002	< 0.001
	L crus II	−24.5, −79.5, −35	5	5.384	0.003	< 0.001
	L superior occipital	−14.5, −99.5, 15	5	5.376	0.003	< 0.001
	L cuneus	−9.5, −92, 27.5	12	5.270	0.006	< 0.001
	L superior frontal	−22, 55.5, 12.5	4	5.262	0.007	0.001
	R crus II	43, −59.5, −45	3	5.255	0.007	0.002
	R inf frontal, pars triangularis	58, 25.5, 0	7	5.171	0.011	< 0.001
	L lingual	−17, −82, −7.5	3	5.157	0.012	0.002
	L middle cingulate	−4.5, −14.5, 37.5	5	5.144	0.013	< 0.001
	L superior temporal	−62, −44.5, 20	3	5.005	0.027	0.002
E > I	L superior frontal (FEF)	−24.5, −4.5, 50	69	6.153	< 0.001	< 0.001
	L superior parietal	−27, −54.5, 55	602	6.476	< 0.001	0.000
	R supramarginal	45.5, −34.5, 42.5	88	6.400	< 0.001	< 0.001
	R angular	23, −62, 47.5	216	6.220	< 0.001	0.000
	R superior frontal (FEF)	28, −2, 55	50	5.648	< 0.001	< 0.001
	L culmen	−24.5, −59.5, −32.5	6	5.430	0.003	< 0.001
	L vermis	−2, −74.5, −22.5	4	5.372	0.004	0.001
	L superior parietal	−29.5, −47, 65	3	5.227	0.008	0.002
	R superior frontal	28, 3, 65	3	5.209	0.009	0.002
	R culmen	35.5, −42, −35	4	4.961	0.033	0.001

*Note:* Results from the second‐level GLM analyses (one‐sample *t* test) testing for the group effects of domain on brain activation. Specific contrasts include: internal versus external domain (all trial types included), external versus internal domain (all trial types included). Results survived a threshold of *p* < 0.05 with family‐wise error (FWE) correction.

Abbreviations: E = external tasks, I = internal tasks.

**FIGURE 5:**
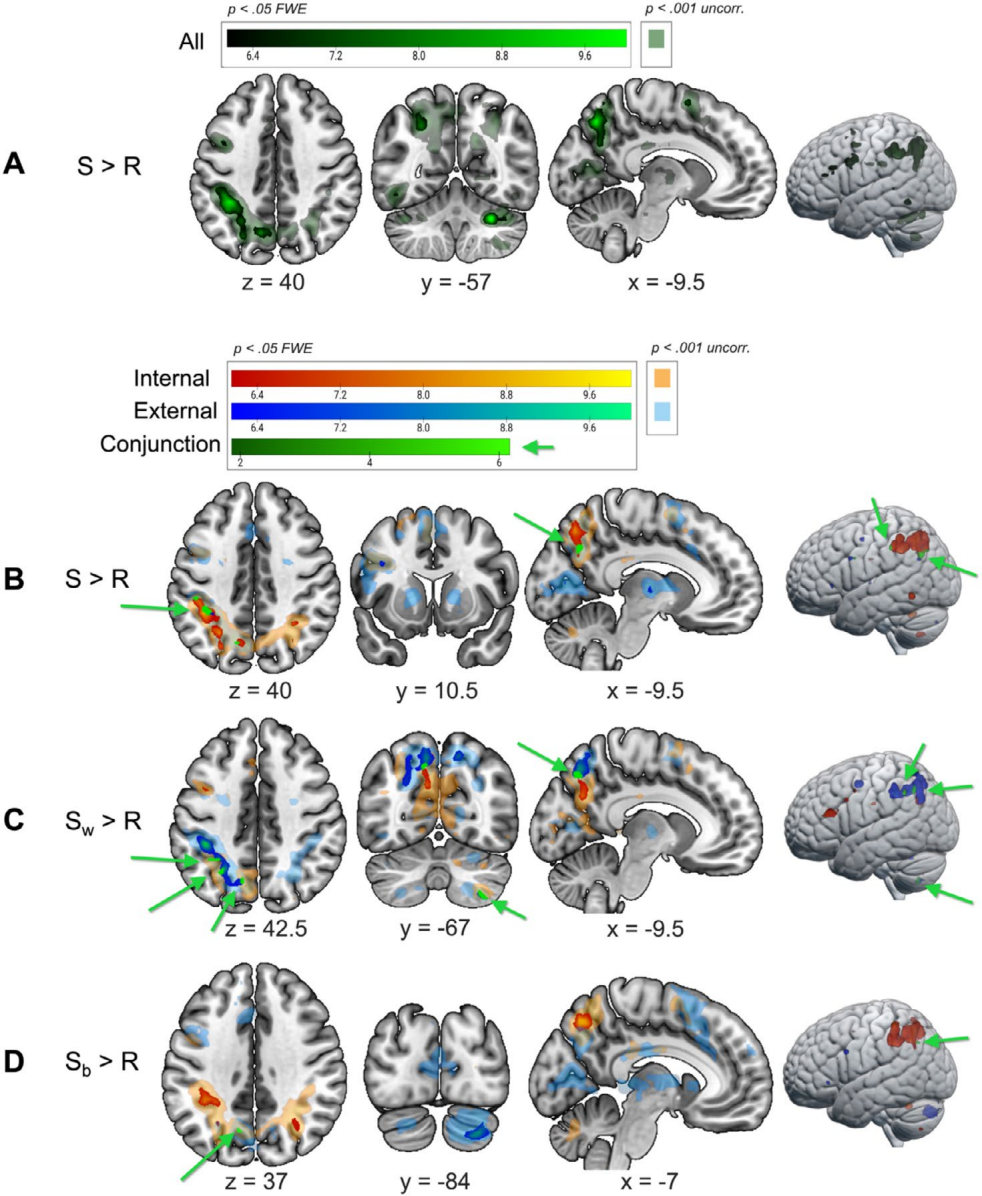
Difference in brain activation between switches and repetitions. Activation at the group level (one‐sample *t* test) for the whole‐brain comparisons between switches and repetitions. Specifically, the first‐level GLMs compared ‘all switches > all repetitions’(A), ‘internal switches > internal repetitions’ and ‘external switches > external repetitions’ (their conjunction performed at the second level is shown in green) (B), ‘switches within internal > internal repetitions’ and ‘switches within external > external repetitions’ (their conjunction performed at the second level is shown in green) (C), ‘switches from external to internal > internal repetitions’ and ‘switches from internal to external > external repetitions’ (their conjunction performed at the second level is shown in green) (D) on a multi‐run model for each subject (four runs of task per participant). The activation maps are shown at *p* < 0.05 FWE‐corrected (bright colours; warm scale = internal; cold scale = external); green scale = both domains together (A) or conjunctions (C, D) and rendered on a standard template (mni152 template in MRIcroGL). Uncorrected activation maps at *p* < 0.001 are also shown for representational purposes with faint, transparent colours. For each slice, the relative MNI coordinate is reported.

Regarding the effects of switching (Figure 5), when comparing the preparation of all switches versus all repetitions, we found left‐lateralised activity in DAN regions (IPL, IPS, precentral gyrus) as well as precuneus, supplementary motor cortex (SMA), inferior frontal lobe, inferior temporal lobe and right cerebellum (Table 3A), in line with Hypothesis 2.

**TABLE 3:** fMRI results on the effects of switching.

Contrast	Region (AAL3)	Peak coordinates	Voxels per cluster	*z*‐score	Peak *p*_FWE‐corr_	Cluster *p*_FWE‐corr_
S > R	R culmen	28, −57, −30	133	6.898	< 0.001	0.000
	L precuneus	−9.5, −69.5, 47.5	167	6.590	< 0.001	0.000
	L inferior parietal lobule	−37, −42, 40	313	6.573	< 0.001	0.000
	R vermis	8, −72, −27.5	30	6.287	< 0.001	< 0.001
	R inferior semilunar lobule	33, −67, −52.5	60	6.044	< 0.001	< 0.001
	L parahippocampal	−19.5 − 39.5, 2.5	3	5.810	< 0.001	0.002
	L precentral	−39.5, 8, −39.5	84	5.726	< 0.001	< 0.001
	L cingulate	−2, −27, 27.5	20	5.647	< 0.001	< 0.001
	L inferior temporal	−52, −52, −10	21	5.592	0.001	< 0.001
	L SMA	−9.5, 3, 62.5	10	5.576	0.001	< 0.001
	L inf frontal, pars triangularis	−42, 25.5, 25	27	5.562	0.001	< 0.001
	L cingulate	−4.5, −17, 27.5	8	5.424	0.003	< 0.001
	L middle frontal	−24.5, 3, 50	17	5.407	0.003	< 0.001
	L inf frontal, pars triangularis	−42, 33, 17.5	10	5.326	0.005	< 0.001
	R sup parietal/precuneus	13, −67, 50	7	5.188	0.010	< 0.001
	R cingulate	8, −19.5, 27.5	3	5.143	0.013	0.002
	L SMA	−4.5, 10.5, 57.5	6	5.025	0.024	< 0.001
SI > RI	R culmen	28, −57, −32.5	22	6.394	< 0.001	< 0.001
	L inferior parietal lobule	−37, −42, 40	294	6.333	< 0.001	0.000
	L precuneus	−7, −69.5, 50	75	5.948	< 0.001	< 0.001
	L inferior temporal	−52, −54.5, −7.5	18	5.722	< 0.001	< 0.001
	R parietal	30.5, −52, 40	26	5.669	< 0.001	< 0.001
	R inferior semilunar lobule	33, −64.5, −52.5	19	5.502	0.002	< 0.001
	L cingulate	−4.5, −12, 27.5	3	5.324	0.005	0.002
	R sup parietal/precuneus	13, −67, 50	16	5.261	0.007	< 0.001
	R parietal	33, −62, 32.5	3	5.076	0.018	0.002
	R superior parietal lobule	30.5, −57, 60	3	5.057	0.020	0.002
	L inferior semilunar lobule	−32, −69.5, −52.5	3	4.985	0.029	0.002
SE > RE	L middle frontal/precentral	−39.5, 10.5, 30	5	5.393	0.003	< 0.001
	L insula	−29.5, 28, 0	5	5.318	0.005	< 0.001
	L ventrolateral thalamus	−9.5, −12, 2.5	3	5.275	0.006	0.002
	L precuneus/cuneus	−14.5, −64.5, 27.5	5	5.203	0.009	< 0.001
	L inferior parietal	−32, −44.5, 40	5	5.197	0.010	< 0.001
	L superior frontal	−24.5, −4.5, 50	5	5.184	0.010	< 0.001
	L precuneus	−12, −64.5, 35	7	5.145	0.013	< 0.001
	R tuber	28, −84.5, −40	5	5.111	0.015	< 0.001
	L mid occipital	−27, −67, 32.5	3	4.907	0.044	0.002
Conjunction	L inf parietal lobule	−37, −42, 40	10	5.568	0.001	< 0.001
SI > RI+	L mid occipital	−27, −64.5, 32.5	14	5.460	0.002	< 0.001
SE > RE	L precuneus	−12, −67, 37.5	12	5.271	0.006	< 0.001
	R culmen	28, −57, −32.5	5	5.216	0.009	0.001
	R inf parietal lobule	−44.5, −32, 42.5	5	5.155	0.012	0.001
S_w_I > RI	L inf frontal, pars triangularis	−47, 28, 20	44	5.911	< 0.001	< 0.001
	L precuneus	−9.5, −67, 30	31	5.688	< 0.001	< 0.001
	L cingulate	−4.5, −17, 27.5	3	5.584	0.001	0.002
	L middle frontal	−39.5, 3, 42.5	5	5.341	0.004	< 0.001
	L middle frontal	−37, 10.5, 35	8	5.245	0.007	< 0.001
	L inf frontal, pars triangularis	−37, 20.5, 22.5	3	5.099	0.016	0.002
	L inferior parietal lobule	−37, −49.5, 40	3	5.013	0.026	0.002
S_w_E > RE	L IPL/precuneus	−39.5, −37, 42.5	341	6.311	< 0.001	0.000
	L middle frontal	−24.5, −2, 50	21	6.127	< 0.001	< 0.001
	R precuneus	15.5, −69.5, 52.5	24	5.932	< 0.001	< 0.001
	R inferior semilunar lobule	33, −67, −52.5	13	5.461	0.002	< 0.001
	R angular	28, −62, 45	5	5.443	0.002	< 0.001
	R declive	25.5, −59.5, −30	6	5.211	0.009	< 0.001
Conjunction	R inferior semilunar lobule	33, −67, −52.5	8	5.539	0.001	< 0.001
S_w_I > RI+	L inferior parietal	−27, −59.5, 42.5	7	5.323	0.005	< 0.001
S_w_E > RE	L precuneus	−12, −67, 42.5	11	5.320	0.005	< 0.001
	L inferior parietal lobule	−34.5, −49.5, 40	9	5.053	0.021	< 0.001
S_b_I > RI	L inferior parietal	−27, −57, 42.5	332	6.236	< 0.001	0.000
	L precuneus	−7, −67, 50	64	6.110	< 0.001	< 0.001
	R culmen	28, −57, −30	16	5.969	< 0.001	< 0.001
	R superior parietal	18, −64.5, 55	53	5.699	< 0.001	< 0.001
	R angular/SPL	30.5, −59.5, 47.5	87	5.487	0.002	< 0.001
	R inferior semilunar lobule	−29.5, −67, −52.5	4	5.457	0.002	0.001
	L superior frontal	−22, 3, 50	3	5.070	0.019	0.002
S_b_E > RE	R crus II	28, −84.5, −37.5	115	6.012	< 0.001	0.000
	Undefined	−24.5, 28, −5	6	5.759	< 0.001	< 0.001
	L precentral/frontal inf opercular	−39.5, 10.5, 30	11	5.613	< 0.001	< 0.001
	R crus II	10.5, −87, −40	4	5.520	0.002	0.001
	R crus II	13, −92, −32.5	5	5.263	0.007	< 0.001
	Undefined	25.5, −92, −25	5	5.038	0.022	< 0.001
Conjunction						
S_b_I > RI+	L Precuneus	−12, −67, 37.5	3	4.994	0.028	0.004
S_b_E > RE						

*Note:* Results from the second‐level GLM analyses (one‐sample *t* test) testing for the group effects of switching on brain activation. Contrasts include: All switches versus all repetitions; switches to internal versus internal repetitions and switches to external versus external repetitions (plus conjunction analysis); switches within internal versus internal repetitions and switches within external versus external repetitions (plus conjunction analysis); external to internal switches versus internal repetitions and internal‐external switches versus external repetitions (plus conjunction analysis). Results survived a threshold of *p* < 0.05 with family wise error (FWE) correction.

Abbreviations: R = all repetitions, RE = repetition of external tasks, RI = repetition of internal tasks, S = all switches, S_b_E = switches from internal to external tasks (between‐domain), S_b_I = switches from external to internal tasks (between‐domain), SE = all switches towards external tasks, SI = all switches towards internal tasks, S_w_E = switches within external tasks, S_w_I = switches within internal tasks.

When focusing on each domain specifically, we found that preparing to switch towards internal tasks (both from the other internal task and from external tasks) elicited greater activation in the above‐mentioned DAN/ECN regions (e.g., left IPL, precuneus, SPL) as compared to repeating internal tasks (Table 3B). Preparing to switch towards external tasks (both from the other external task and from internal tasks) instead resulted in greater activation in small clusters (< 10 voxels) of the DAN but also SN network (left middle frontal and precentral gyrus, left insula, precuneus and IPL) as compared to external task repetitions (Table 3C). The conjunction analysis of these two contrasts (all switches towards internal > repetitions of internal + all switches towards external > repetitions of external) showed a joint activation of left IPL, middle occipital cortex and precuneus (Table 3D), partially aligning with Hypothesis 3 although no activation in the MD thalamus was found (see Figure S2 and Table S5) for the results of the opposite contrasts (repetitions vs. switches in each domain).

We performed a more detailed examination of switch costs by focusing on each switch type as well as each domain. Switches within the internal domain elicited greater preparatory activation in the left inferior frontal lobe (pars triangularis) and DMN precuneus, as compared to repetitions of internal tasks (Table 3E). Switches within the external domain elicited greater activation in DAN regions (left IPL and middle frontal lobe), bilateral precuneus and right cerebellum when compared to repetitions of external tasks (Table 3F). Interestingly, the conjunction analysis showed a joint activation of the left precuneus by these two contrasts (switches within internal > repetitions of internal + switches within external > repetitions of external) (Table 3G). Instead, the preparation of switches from external to internal tasks resulted in greater activation of some DAN regions (left IPL), bilateral precuneus and cerebellum as compared to repetitions of internal tasks (Table 3H). In turn, switches from internal to external tasks induced activation in right cerebellum and left precentral gyrus as compared to repetitions of external tasks (Table 3I). The conjunction analysis of these two contrasts (external–internal switches > repetitions of internal + internal‐external switches > repetitions of external) showed a joint activation of a different portion of the left precuneus (although only a small cluster of three voxels) with respect to that activated by within‐domain switches (Table 3J).

**FIGURE 6:**
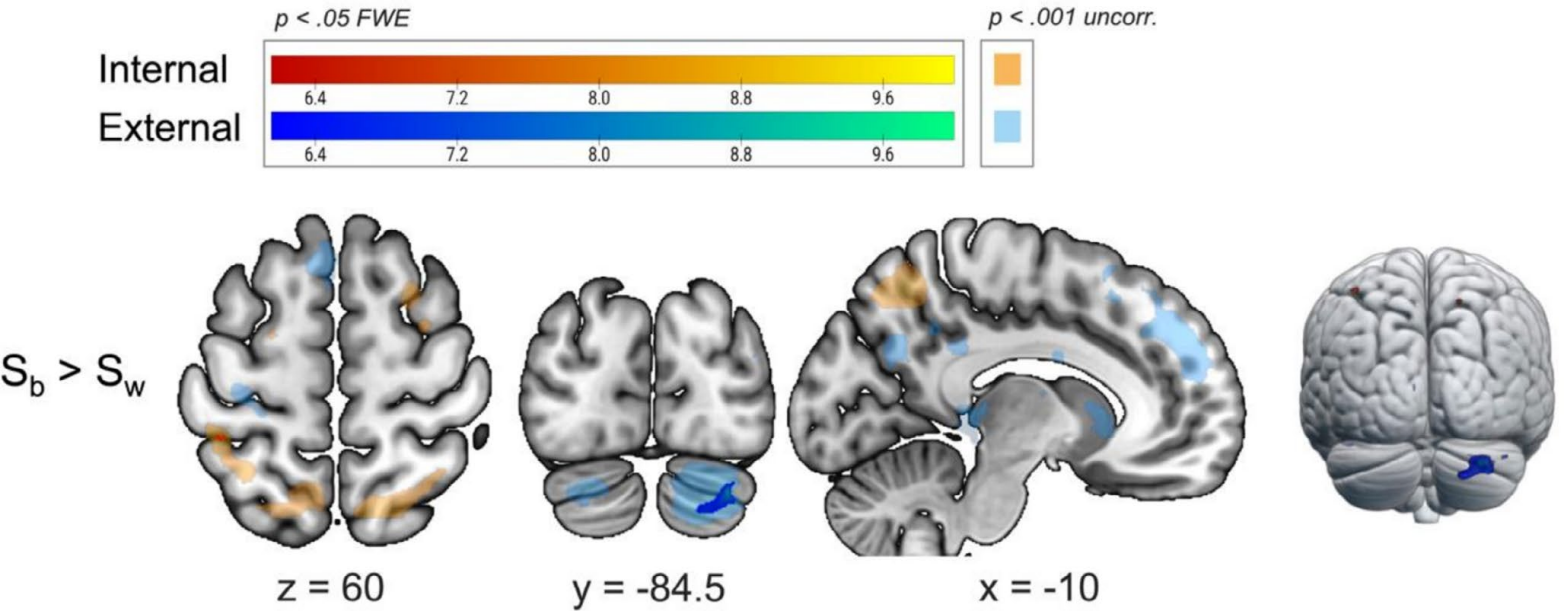
Difference in brain activation between switch types. Brain activation at the group level (one‐sample *t* test) for the whole‐brain comparisons between switch types. Specifically, the first‐level GLMs compared ‘switches from external to internal > switches within internal’ and ‘switches from internal to external > switches within external’ on a multi‐run model for each subject (four runs of task per participant). The activation maps are shown at *p* < 0.05 FWE‐corrected (bright colours; warm scale = internal; cold scale = external) and rendered on a standard template (mni152 template in MRIcroGL). Uncorrected activation maps at *p* < 0.001 are also shown for representational purposes with faint, transparent colours. For each slice, the relative MNI coordinate is reported.

Finally, we investigated the differences between switch types (Figure 6). No voxels survived the multiple comparisons correction when comparing all between‐domain switches versus all within‐domain switches, against Hypothesis 4. Looking at the two domains separately, we found that switching from external to internal tasks elicited greater activation in DAN (left IPL and right SPL) compared to switching within internal tasks (small clusters of four and three voxels only, respectively; Table 4A). Instead, switches from internal to external tasks induced greater preparatory activation mostly in the right cerebellum, as compared to switches within external tasks (Table 4B). No voxels survived correction in the conjunction analysis between the two latter contrasts.

**TABLE 4:** fMRI results on the differences between switch types.

Contrast	Region (AAL3)	Peak coordinates	Voxels per cluster	*z*‐score	Peak *p*_FWE‐corr_	Cluster *p*_FWE‐corr_
S_b_I > S_w_I	L inf parietal lobule/postcentral	−39.5, −39.5, 60	4	5.628	0.001	0.001
	R superior parietal lobule	18, −67, 57.5	3	5.233	0.008	0.002
S_b_E > S_w_E	R crus I (uvula)	30.5, −84.5, −32.5	91	6.019	< 0.001	< 0.001
	L inferior temporal	−42, 5.5, −35	8	5.191	0.010	< 0.001
	L posterior cingulate	−7, −37, 5	4	5.188	0.010	0.001

*Note:* Results from the second‐level GLM analyses (one‐sample *t* test) testing for the group differences between switch types. Contrasts include: switches from external to internal versus switches within the internal domain; switches from internal to external versus switches within the external domain. Results survived a threshold of *p* < 0.05 with family‐wise error (FWE) correction.

Abbreviations: S_b_E = switches from internal to external tasks (between‐domain), S_b_I = switches from external to internal tasks (between‐domain), S_w_E = switches within external tasks, S_w_I = switches within internal tasks.

Across all contrasts on the effect of switching, applying small volume correction over the bilateral thalamus (AAL mask) did not reveal any further thalamic involvement, beyond the small activation (three voxels) found in the ventrolateral thalamus during preparation of switches to external tasks versus repetitions of external tasks.

Following Reviewers' suggestions, to rule out a possible effect of motor preparation and sensory processing in the cue to target phase, we repeated the above GLMs including an additional regressor of no interest covering the period from stimulus onset to button press. This yielded largely consistent findings (see Tables S6–S8 and Figures S3–S6). While a small number of peaks no longer survived FWE correction, this is likely due to reduced statistical power related to model overfitting (Soch et al. 2016), as our paradigm and GLMs were designed to subtract any potential sensory or motor preparation effects by ensuring our stimuli (both geometric cues and adjectives) and required responses (button press) were equivalent across conditions.

### 3.3 DCM Results

On average, individual DCM models explained 19.23% of variance (SD = ±9.37). This is unsurprising and acceptable in the case of task designs in which baseline periods (e.g., ITIs) are not modelled (Zeidman, Jafarian, Corbin, et al. 2019).

**FIGURE 7:**
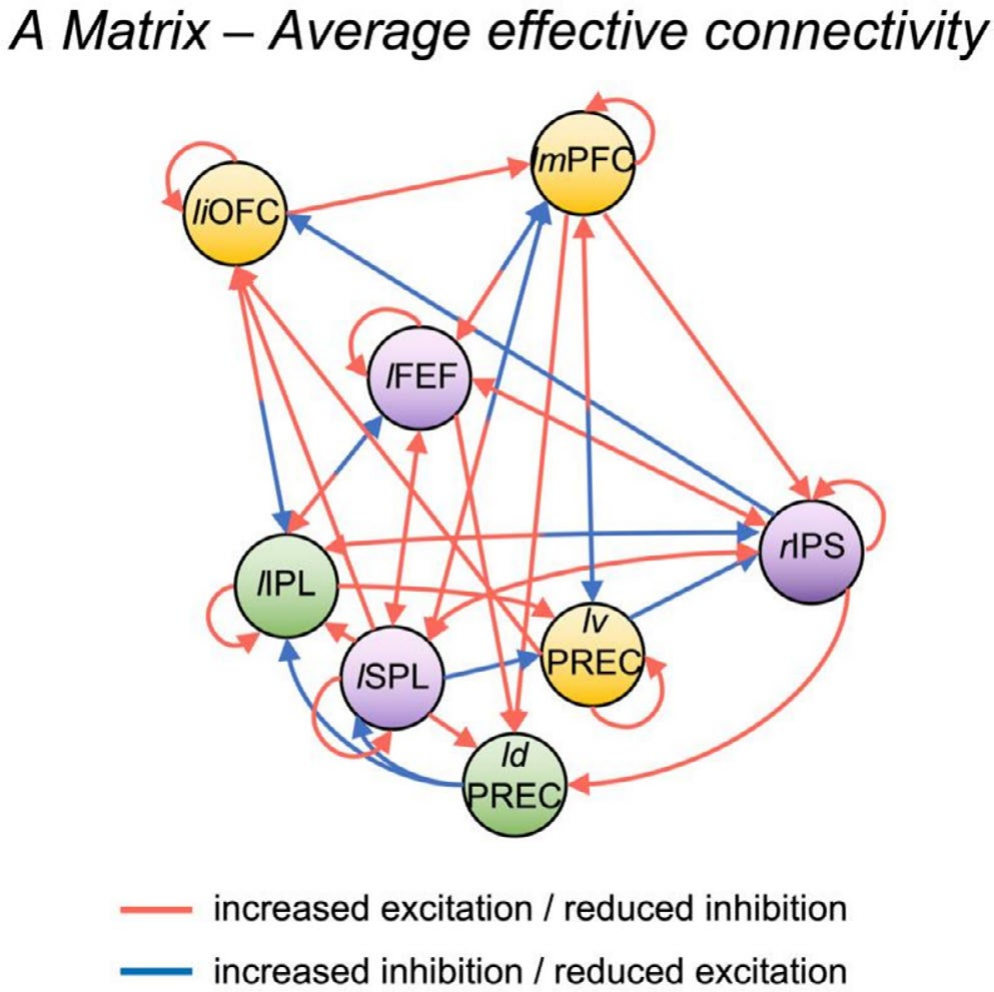
Estimated average effective connectivity across experimental conditions (A matrix). We report the connections that exceeded the 95% posterior probability threshold in the PEB analysis. Red arrows indicate more excitation/less inhibition as compared to baseline (i.e., rest), while blue arrows signal connections with more inhibition/less excitation compared to baseline. Note that self‐connections are always inhibitory so red indicates a reduction in inhibition while blue indicates increased inhibition. The coordinates of yellow regions were extracted from the GLM contrast ‘internal > external’, those of purple regions were extracted from ‘external > internal’ and those of green regions were extracted from ‘all switches > all repetitions’. Abbreviations: *ld*PREC = left dorsal precuneus; *l*FEF = left frontal eye fields; *li*OFC = left inferior orbitofrontal cortex; *l*IPL = left inferior parietal lobule; *lm*PFC= left medial prefrontal cortex; *l*SPL = left superior parietal lobule; *lv*PREC = left ventral precuneus; rIPS = right intraparietal sulcus.

At the group level, the estimation of average effective connectivity across conditions (A matrix) highlighted a pattern of excitation between regions of the DAN, such as between SPL, IPS and FEF bidirectionally, as well as from those regions to dorsal precuneus and IPL, accompanied by inhibition from DAN towards DMN regions (Figure 7). Interestingly, we also found that IPL and dorsal precuneus showed excitatory effective connectivity towards DMN regions and inhibitory effective connectivity towards the other DAN regions.

**FIGURE 8:**
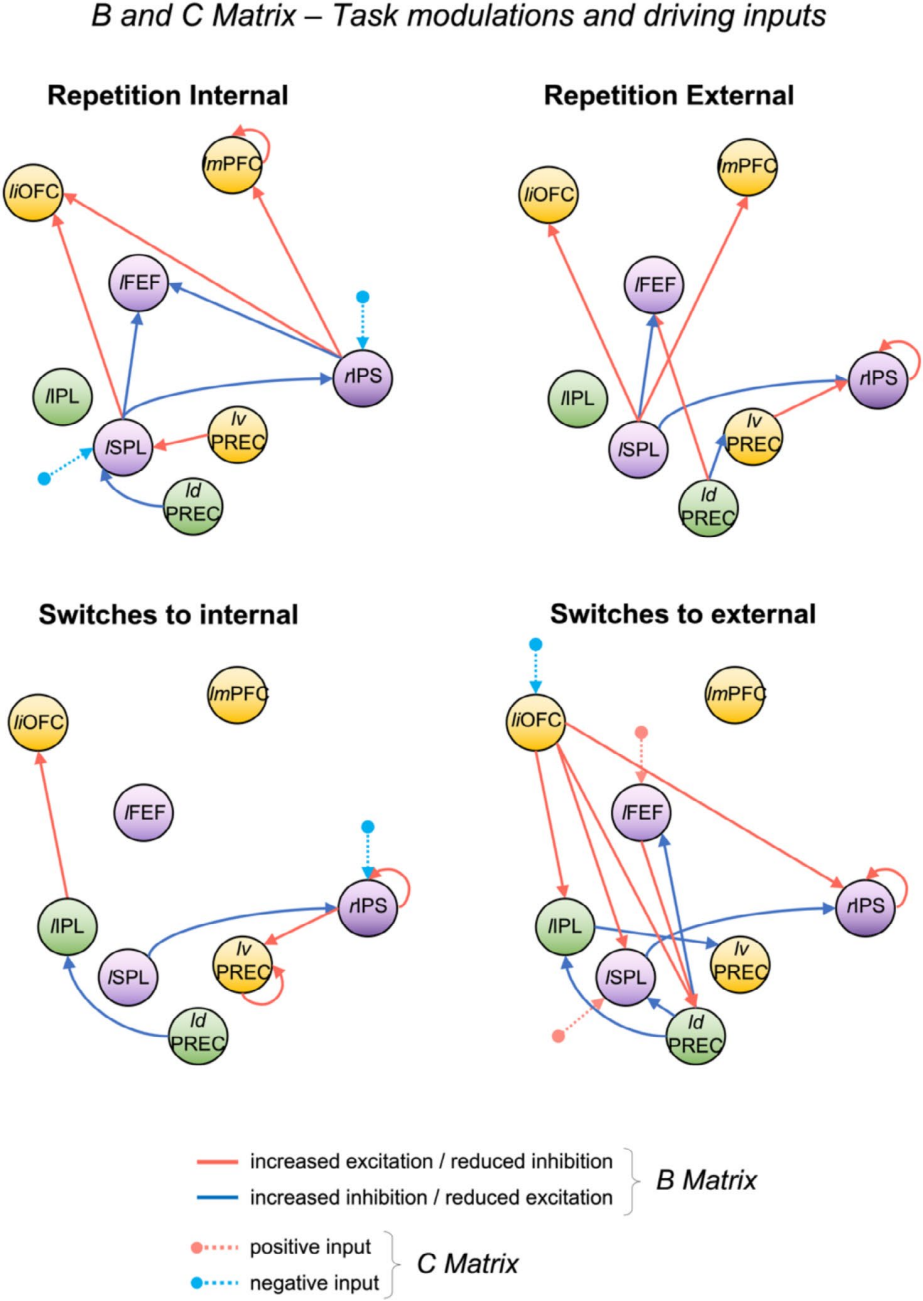
Effects of task conditions on the network's effective connectivity. We report the connections that exceeded the 95% posterior probability threshold in the PEB analysis. Solid lines represent the modulatory effect of each task condition (B matrix) while dotted lines indicate the driving inputs from the task (C matrix)—that is, where each task condition enters the network. Red solid lines indicate an excitatory modulation (up‐regulation) by a specific task condition, while blue lines indicate inhibitory modulation (down‐regulation). In the case of self‐connections, the modulation is interpreted as decreased (red) or increased (blue) inhibition, which means self‐connections become more or less sensitive (respectively) to inputs from the rest of the network as a result of task influence. Light red dotted lines indicate a positive drive from task condition while light blue dotted lines indicate a negative drive. The coordinates of yellow regions were extracted from the GLM contrast ‘internal > external’, those of purple regions were extracted from ‘external > internal’ and those of green regions were extracted from ‘all switches > all repetitions’. Abbreviations: *ld*PREC = left dorsal precuneus; *l*FEF = left frontal eye fields; *li*OFC = left inferior orbitofrontal cortex; *l*IPL = left inferior parietal lobule; *lm*PFC = left middle prefrontal cortex; *l*SPL = left superior parietal lobule; *lv*PREC = left ventral precuneus; RE = external repetitions; RI = internal repetitions; rIPS = right intraparietal sulcus; SWE = switches to external; SWI = switches to internal.

The estimation of driving inputs (C matrix; dotted lines in Figure 8) indicated that the preparation of internal repetitions perturbed the network via a negative drive into two DAN regions (right IPS and left SPL). Similarly, the preparation of switches towards internal tasks negatively perturbed the right IPS. On the contrary, preparing for switches towards external tasks negatively perturbed the inferior OFC and positively affected left SPL and FEF. No driving inputs surpassed the 95% posterior probability threshold in the external repetitions condition.

The estimation of modulatory inputs (B matrix; solid lines in Figure 8) showed that the preparation of internal task repetitions modulated the connectivity from both precuneal subregions towards the left SPL (inhibition by dorsal and excitation by ventral precuneus), which in turn inhibited the connectivity towards left FEF and right IPS and excited the left iOFC. The right IPS also reduced its inhibition towards iOFC, excited mPFC (which showed increased sensitivity to inputs) and inhibited the left FEF. Instead, the preparation of external task repetitions induced inhibition from dorsal to ventral precuneus, which in turn excited the right IPS (displaying increased sensitivity to inputs). The dorsal precuneus also excited the left FEF. Most of the modulations were, however, located in the effective connectivity from left SPL towards iOFC, mPFC (both excited), FEF and IPS (inhibited). The preparation of switches to the internal domain instead induced inhibition from the dorsal precuneus towards left IPL, which then excited iOFC. Both left ventral precuneus and right IPS displayed increased sensitivity to inputs, the right IPS receiving inhibition from the left SPL and in turn exciting the ventral precuneus. Finally, the preparation of switches to the external domain elicited widespread excitatory modulations from iOFC to all DAN regions (except FEF). Inhibitory modulations were found from dorsal precuneus to left SPL, IPL and FEF. The left SPL in turn inhibited the right IPS, while the left IPL inhibited the ventral precuneus.

Table S9 reports the group coordinates of the eight ROIs included in the DCM analysis. The values of all estimated parameters are also reported in Figures S7–S9 (for A, B and C matrices, respectively).

## 4 Discussion

In this study, we collected fMRI data while participants performed a cued task‐switching paradigm with two internally oriented and two externally oriented tasks and investigated the BOLD activity associated with anticipatory preparation of switches within and between the internal and external domains. We found that switching is accompanied by slower RTs and the anticipatory activation of lateral frontoparietal regions in both the internal and external domains, as compared to repetitions. In addition, we observed DMN activation in preparation for internal tasks.

First, we replicated the main behavioural results from our previous study (Calzolari et al. 2022). Specifically, we observed again a switch cost effect both in the external and internal domains, as well as an interaction between domain and trial type. We previously interpreted our behavioural results as evidence for the presence of two domain‐specific cognitive systems that communicate with each other and share domain‐general features to allow the flexible shift to a different task and/or domain (Calzolari et al. 2022). Our fMRI results confirm this view. First, when comparing the preparation of internal versus external tasks regardless of trial type, we found activation in regions of the core DMN system associated with self‐reflection and autobiographical memory, as well as regions of the extended dorsal medial PFC subsystem associated with social reasoning and mentalizing (Andrews‐Hanna et al. 2010). Some of these areas, such as the OFC and temporal lobe, are also associated with language comprehension, and in particular with controlled semantic retrieval and semantic judgement (Abutalebi et al. 2008; Sabb et al. 2007), which is expected due to the nature of the internal tasks in our paradigm. In contrast, when comparing external versus internal trials, we found activation in areas of the DAN bilaterally (Szczepanski et al. 2013). In accordance with this, our DCM results show an overall pattern of excitatory effective connectivity within DAN areas and within DMN areas, but an inhibitory effective connectivity from DAN areas towards DMN. Taken together, these results are in line with previous research suggesting a role of DMN areas in the processing of various types of self‐referential information and implicating the DAN in visuospatial attention (Braga et al. 2013; Corbetta and Shulman 2002; Miller and Buschman 2013). However, it is important to note that our results demonstrate the involvement of these networks in the preparatory phase of internally and externally oriented tasks, prior to the actual task execution and prior to viewing the stimulus. While previous studies have shown the presence of a rapid, preparatory engagement of task‐specific areas during switch cueing (Murray et al. 2009; Wylie et al. 2006), the fact that the DMN also displays such task‐driven activation, in particular, is noteworthy.

A previous study investigating switches between task and rest found rapid modulation of DMN regions during cue‐to‐target intervals (Sidlauskaite et al. 2014). Specifically, DMN activation (lingual gyrus [−18, −74, −12], superior medial frontal gyrus [13, 63, 20] and precuneus [−12, −49, 41]) increased after cues signalling upcoming rest and decreased after rest‐to‐task cues. Conversely, the anterior insula [−29, 24, 10] activated in anticipation of task performance when switching from rest to task. This supports the idea that anticipatory preparation might be flexibly assigned to different networks depending on the required brain state and specifically demonstrates an early engagement of DMN in preparation of a task‐related instruction change (Sidlauskaite et al. 2014). Our study expands our understanding of the role of the DMN in identifying that a similar activation pattern is also present during the preparation of self‐referential tasks in a cognitive switching context. Indeed, we demonstrate that the DMN is actively involved in the goal‐directed control of (internally oriented) cognition and that its connectivity pattern with DAN can flexibly adjust based on task requirements. While, as stated above, the majority of DAN regions presented excitatory connections within the network and inhibitory effective connectivity towards DMN, the left IPL and dorsal precuneus (which we found engaged during task switching) instead displayed inhibitory effective connectivity towards other DAN regions and excitatory effective connectivity towards DMN. Moreover, the effective connectivity modulations induced by the task revealed that DMN regions upregulated regions of the DAN in preparation of switching towards the external domain. These results discredit the idea of DMN as a purely ‘task‐negative’ or even ‘stimulus‐independent’ network (Anticevic et al. 2012). The growing number of studies finding an active role of DMN in cognition suggests that this network is involved in the representation of context and situations (both current and internally generated or retrieved from memory), a view that could merge and explain the manifold range of DMN functions (Smith et al. 2018; Spreng 2012).

During the preparation of switch trials, we observed a pattern of activation that partially overlapped with the set of areas we found active in preparation of external (vs. internal) tasks (DAN network), although peaking in more ventral and lateral portions. Specifically, a wide cluster in the left IPL/IPS as well as left superior frontal/precentral gyrus (including the IFJ) was more active in preparation of switches as compared to repetitions. This is in line with previous studies demonstrating left lateralization of brain activity associated with task‐switching (Badre and Wagner 2006; Kim et al. 2011; Vallesi et al. 2015) as well as with semantic and lexical processing in general (Braver et al. 2003). Further, these results are mostly consistent with previous task‐switching studies showing greater activity in lateral frontoparietal regions during switches as compared to repetitions (Crone et al. 2006; Jamadar et al. 2010; Vallesi et al. 2015; Witt and Stevens 2013), although the current patterns show a larger involvement of parietal DAN regions as compared to frontal ECN/SN regions (e.g., dorsolateral PFC, dorsal ACC) (Brass and von Cramon 2004). The activation of IPL and IPS has been implicated in visuospatial reorientation and working memory, but also with task‐rule representation and mapping of relevant responses during cued task‐switching (De Baene and Brass 2014; Fox et al. 2005; Natale et al. 2008). This difference with previous literature might be thus due to the adoption of long preparation intervals (CTI), which naturally favour the implementation of working memory processing to aid task‐switching (Brass and von Cramon 2004) and likely attenuates the activity in conflict monitoring/detection areas (e.g., ACC and pre‐SMA, showing small activation clusters when correcting for multiple comparisons, as per Figure 5A) (Walsh et al. 2011). However, although smaller than parietal clusters, we still observed clusters of activation in portions of the inferior frontal gyrus, which have been consistently linked with general goal‐shifting and general task representations (Brass and von Cramon 2004).

In line with these results, our DCM analysis on task‐induced modulations showed that switching preparation in both domains modulates widespread connectivity between parietal areas (or from frontal to parietal regions). In contrast, the preparation of task repetitions appeared to target frontal regions instead (i.e., modulating connections from parietal towards frontal regions). This may indicate that, during task repetitions, more frontal regions are involved in the maintenance of current task rules while, during switching, the parietal cortex is engaged in the retrieval and reconfiguration of currently relevant action sets (Philipp et al. 2013). Notably, the driving inputs of the repetitions of internally oriented tasks involved inhibitory modulations to both IPS and lSPL, whereas switches to internally oriented tasks only showed inhibitory input to IPS, suggesting that task repetition requires broader suppression of DAN regions. The additional inhibition of SPL during task repetitions may reflect reduced demands for cognitive control processes typically required for task set reconfiguration (Sohn et al. 2000). In addition, preparation of switches towards internal tasks resulted in excitatory connections from parietal to DMN and language regions (ventral precuneus and iOFC). Instead, switches to the external domain mostly induced an excitatory modulation from DMN regions (the iOFC) towards all DAN parietal regions. This clearly highlights the presence of dynamic patterns of connectivity where frontoparietal networks flexibly reorganise based on domain and task demands.

Importantly, we also found a marked activation of the left dorsal precuneus during switch preparation. This portion of the precuneus is thought to be part of a domain‐general frontoparietal control network implicated in a range of executive functions (Niendam et al. 2012), and is generally associated with working memory/category encoding (Braunlich et al. 2015) and visuospatial reorienting (Indovina and Macaluso 2007). In agreement with our results, a number of task‐switching studies also found activation in this dorsal portion of precuneus extending towards the SPL, as well as in the IFJ, during task switches (vs. repetitions) regardless of task rule and/or domain, confirming the important role of these areas in a superordinate, general type of cognitive control (Chiu and Yantis 2009; Stelzel et al. 2011; Yeung et al. 2006). The precuneus is a highly heterogeneous region and its subregions are characterised by different functional connectivity (FC) patterns at rest, both in humans and macaque monkeys (Cauda et al. 2010; Margulies et al. 2009). Margulies and colleagues identified a ‘central’ precuneal region [−5, −66, 44] connected to IPL and dorsal PFC and thus associated with high‐order executive processing and working memory. In the same study, a more ventral subregion [−2, −59, 36] was connected to DMN regions (medial PFC, medial temporal cortex) and hippocampal/parahippocampal cortices (Margulies et al. 2009). Similarly, others also found differential resting‐state FC patterns for dorsal and ventral precuneus, which were functionally connected to the parietal cortex and posterior cingulate/mPFC, respectively (Zhang and Li 2012). Given these heterogeneous patterns, some researchers suggested a pivotal role of the precuneus in connecting and interfacing distinct frontoparietal networks that subserve internal and external processing (Leech et al. 2011; Lyu et al. 2021). Our results support this view by observing a strong involvement of the dorsal precuneus in task switching and an engagement of ventral precuneus in preparation of internal tasks. Importantly, in our study, the precuneus is the only region that displays conjoint activation across all types of switches (within and between domains), further confirming its role in domain‐general cognitive control.

We also replicated the main effect of switch type which would indicate additional costs for switching between domains compared to within domain. However, this effect was not present in the post hoc tests that measured costs for the external and internal domains separately. Since the Bayesian evidence did not confirm the absence of a behavioural effect, we believe this lack of significance might be due to the smaller sample size in the current fMRI study (*n* = 30 here vs. *n* = 200 previously), and the small number of trials that contribute to this particular comparison as compared to the main effects and interactions, which (as discussed above and earlier on in this paper) did replicate. While it is not possible to rule out that the scanner environment may have affected task performance compared to our first study, it is nevertheless reassuring that the main behavioural effects reported in an online study replicated in a controlled lab environment. Similarly, the only noticeable difference between switch types in our fMRI analysis was a greater activation in the right cerebellum when comparing internal‐to‐external switches with switches within external tasks. This again suggests that the number of trials might have been insufficient to fully capture the differences across switch types. Similarly, and contrary to what we expected, we did not observe thalamic activation during switching, except for a small cluster in the ventrolateral thalamus only for switches in the external domain. Activation in this specific nucleus has been linked to attentional biases towards contents in working memory (de Bourbon‐Teles et al. 2014). Therefore, this partially hints at the presence of thalamic involvement in switching, although it does not show a domain‐general involvement of the MD nucleus. Several technical factors impact the detection of thalamic activation, including lower temporal signal‐to‐noise ratio in the centre of the brain and the generally smaller percent signal change of the BOLD response in subcortical regions compared to the cortex (Forstmann et al. 2017; Risk et al. 2021). Further research is therefore needed to specifically test the role of MD thalamus in cognitive switching.

It is worth mentioning that, despite this being a common design choice (Kiesel et al. 2010), the absence of a neutral baseline condition means it is not possible to reliably disentangle the benefits of task repetition from the costs of task switching (i.e., the same contrast can be interpreted as reduced brain activation in repeat trials or increased activation in switch trials). However, both mechanisms would predict similar patterns of brain activation, and our study was not designed to contrast between these two mechanisms. Previous studies have focused on assessing and discussing this differentiation (e.g., De Baene et al. 2012) with externally oriented tasks, and our study may provide the basis for further research to clarify any potential difference in these mechanisms related to internally versus externally oriented cognition. In addition, we note that some of the parietal ROIs used for our DCM analyses had a mild overlap in a subset of participants due to the proximity of their peak coordinates and the 5 mm FWHM smoothing applied during preprocessing. Specifically, these were SPL‐precuneus (43.3% of subjects), SPL‐IPL (30.0% of subjects) and angular gyrus‐IPL (10.0% of subjects). We considered that the ROI spheres overlapped if the Euclidean distance between the centre peaks was below a conservative threshold of 20.5 mm (twice the sum of the 6 mm extraction radius and the smoothing kernel extent of 2*σ*). While we cannot rule out that this may have affected our DCM results, the relatively large mean distances (23–29 mm) indicate minimal signal sharing, and our extraction procedure included adjustments to reduce spatially correlated noise.

## 5 Conclusions

We employed a cued task‐switching paradigm to directly investigate network reconfiguration during changes in internally and externally oriented cognition. Our findings suggest that the adaptive communication between DMN and DAN can guide task performance in the context of a goal‐directed control of self‐referential and external processing. These dynamics appear supported by the domain‐general activation of dorsal precuneus and IPL.

## Author Contributions

**Sara Calzolari:** conceptualisation, methodology, investigation, data curation, formal analysis, visualisation, writing – original draft, writing – review and editing. **Brandon T. Ingram:** investigation, writing – review and editing. **Andrew P. Bagshaw:** supervision, funding acquisition, writing – review and editing. **Davinia Fernández‐Espejo:** supervision, funding acquisition, conceptualisation, methodology, visualisation, formal analysis, writing – review and editing.

## Supporting information


**Table S1:** List of all GLM pairwise contrasts.
**Table S2:** One sample *t* tests over switch types after subtracting repetitions to check that all switch types in the 2 × 2 rANOVA were significantly different from task repetitions (=0).
**Table S3:**. Descriptive statistics (mean and SD) of RTs for all conditions in the 2 × 2 rANOVA (domain × switch type).
**Table S4:**. Results from the second‐level GLM analyses (one‐sample *t* test) testing for the group effects of domain on brain activation. Specific contrasts include: internal repetitions vs. external repetitions, external repetitions versus internal repetitions, switches to internal versus switches to external, switches to external vs. switches to internal. Results survived a threshold of *p* < 0.05 with family wise error (FWE) correction. Abbreviations: RE = repetition of external tasks; RI = repetition of internal tasks; SE = all switches towards external tasks; SI = all switches towards internal tasks.
**Table S5:** Results from the second‐level GLM analyses (one‐sample *t* test) testing for the group effects of task repetition vs. task switching on brain activation. Specific contrasts include: internal repetitions versus switches to internal, external repetitions vs. switches to external. Results survived a threshold of *p* < 0.05 with family wise error (FWE) correction. Abbreviations: RE = repetition of external tasks; RI = repetition of internal tasks; SE = all switches towards external tasks; SI = all switches towards internal tasks.
**Table S6:** fMRI results on the effects of domain—additional regressor analysis. Results from the second‐level GLM analyses (one‐sample *t* test) testing for the group effects of domain on brain activation, including an additional regressor of no interest (between stimulus onset and button press) to further control for the effect of motor preparation. Specific contrasts include: internal versus external domain (all trial types included), external versus internal domain (all trial types included). Results survived a threshold of *p* < 0.05 with family wise error (FWE) correction. Abbreviations: E = external tasks; I = internal tasks.
**Table S7:** fMRI results on the effects of switching and differences between switch types—additional regressor analysis. Results from the second‐level GLM analyses (one‐sample *t* test) testing for the group effects of switching on brain activation, including an additional regressor of no interest (between stimulus onset and button press) to further control for the effect of motor preparation. Contrasts include: all switches versus all repetitions; switches to internal versus internal repetitions and switches to external versus external repetitions; switches within internal versus internal repetitions and switches within external versus external repetitions; external to internal switches versus internal repetitions and internal‐external switches versus external repetitions. Results survived a threshold of *p* < 0.05 with family wise error (FWE) correction. Abbreviations: R = all repetitions; RE = repetition of external tasks; RI = repetition of internal tasks; S = all switches; S_b_E = switches from internal to external tasks (between‐domain); S_b_I = switches from external to internal tasks (between‐domain); E = all switches towards external tasks; SI = all switches towards internal tasks; S_w_E = switches within external tasks; S_w_I = switches within internal tasks.
**Table S8:** fMRI results on the differences between switch types—additional regressor analysis. Results from the second‐level GLM analyses (one‐sample *t* test) testing for the group differences between switch types, including an additional regressor of no interest (between stimulus onset and button press) to further control for the effect of motor preparation. Contrasts include: switches from external to internal versus switches within the internal domain; switches from internal to external vs. switches within the external domain. Results survived a threshold of *p* < 0.05 with family wise error (FWE) correction. Abbreviations: S_b_E = switches from internal to external tasks (between‐domain); S_b_I = switches from external to internal tasks (between‐domain); S_w_E = switches within external tasks; S_w_I = switches within internal tasks.
**Table S9:** Difference in brain activation between domains. These coordinates were extracted from GLM T‐contrasts and formed the starting point for the search of individual coordinates in the GLM activation maps. Orbitofrontal, ventral precuneus and medial superior frontal cortex were extracted from the contrast ‘internal > external’; superior parietal, supramarginal and lateral superior frontal were extracted from ‘external > internal’; finally, IPL and dorsal precuneus were extracted from ‘switches > repetitions’ (all spheres of 6 mm radius).
**Figure S1:**. Brain activation at the group level (one‐sample *t* test) for the whole‐brain comparisons between domains for each trial type. Specifically, the first‐level GLMs compared ‘internal > external’ and ‘external > internal’ for both repetitions only (A) and switches only (B) on a multi‐run model for each subject (four runs of task per participant). The activation maps are shown at *p* < 0.05 FWE‐corrected (bright colours; warm scale = internal > external; cold scale = external > internal) and rendered on a standard template (mni152 template in MRIcroGL). Uncorrected activation maps at *p* < 0.001 are also shown for representational purposes with faint, transparent colours. For each slice, the relative MNI coordinate is reported.
**Figure S2:** Brain activation at the group level (one‐sample *t* test) for the whole‐brain comparisons between repetitions and switches for each domain type. Specifically, the first‐level GLMs compared ‘internal repetitions > switches to internal’ and ‘external repetitions > switches to external’ on a multi‐run model for each subject (four runs of task per participant). The activation maps are shown at *p* < 0.05 FWE‐corrected (bright colours; warm scale = internal domain; cold scale = external domain) and rendered on a standard template (mni152 template in MRIcroGL). Uncorrected activation maps at *p* < 0.001 are also shown for representational purposes with faint, transparent colours. For each slice, the relative MNI coordinate is reported.
**Figure S3:** Difference in brain activation between GLM versions—Effect of domain. Activation at the group level (one‐sample *t* test) for the whole‐brain comparisons between domains, regardless of trial type. In both versions, the first‐level GLMs compared ‘internal > external’ and ‘external > internal’ on a multi‐run model for each subject (four runs of task per participant). The main GLM (red) only included the button response as regressor of no interest, while the alternative GLM (green) also included a regressor for motor preparation (period between stimulus onset and button press). The uncorrected activation maps are shown at *p* < 0.001 and rendered on a standard template (mni152 template in MRIcroGL). For each slice, the relative MNI coordinate is reported.
**Figure S4:** Difference in brain activation between GLM versions—Effect of switches versus repetitions. Activation at the group level (one‐sample *t* test) for the whole‐brain comparisons between switches and repetitions. In both versions, the first‐level GLMs compared ‘all switches > all repetitions’, ‘internal switches > internal repetitions’ and ‘external switches > external repetitions’ on a multi‐run model for each subject (4 runs of task per participant). The main GLM (red) only included the button response as regressor of no interest, while the alternative GLM (green) also included a regressor for motor preparation (period between stimulus onset and button press). The uncorrected activation maps are shown at *p* < 0.001 and rendered on a standard template (mni152 template in MRIcroGL). For each slice, the relative MNI coordinate is reported.
**Figure S5:** Difference in brain activation between GLM versions—Effect of specific switch types versus repetitions. Activation at the group level (one‐sample *t* test) for the whole‐brain comparisons between specific switch types and repetitions. In both versions, the first‐level GLMs compared ‘switches within internal > internal repetitions’, ‘switches within external > external repetitions’, ‘switches from external to internal > internal repetitions’ and ‘switches from internal to external > external repetitions’ on a multi‐run model for each subject (four runs of task per participant). The main GLM (red) only included the button response as regressor of no interest, while the alternative GLM (green) also included a regressor for motor preparation (period between stimulus onset and button press). The uncorrected activation maps are shown at *p* < 0.001 and rendered on a standard template (mni152 template in MRIcroGL). For each slice, the relative MNI coordinate is reported.
**Figure S6:** Difference in brain activation between GLM versions—Comparison between switch types. Activation at the group level (one‐sample *t* test) for the whole‐brain comparisons between switch types. In both versions, the first‐level GLMs compared ‘switches from external to internal > switches within internal’ and ‘switches from internal to external > switches within external’ on a multi‐run model for each subject (four runs of task per participant). The main GLM (red) only included the button response as regressor of no interest, while the alternative GLM (green) also included a regressor for motor preparation (period between stimulus onset and button press). The uncorrected activation maps are shown at *p* < 0.001 and rendered on a standard template (mni152 template in MRIcroGL). For each slice, the relative MNI coordinate is reported.
**Figure S7:**. Estimated average effective connectivity across experimental conditions (A matrix). We report the connections that exceeded the 95% posterior probability threshold in the PEB analysis. Warm colours in the extrinsic connections indicate more excitation as compared to baseline (i.e., rest), cold colours signal more inhibition compared to baseline. Note that self‐connections are log‐scaled and always inhibitory, so warm colours indicate a reduction in inhibition and cold colours indicate increased inhibition. Abbreviations: *ld*PREC = left dorsal precuneus; *l*FEF = left frontal eye fields; *li*OFC = left inferior orbitofrontal cortex; *l*IPL = left inferior parietal lobule; *lm*PFC = left middle prefrontal cortex; *l*SPL = left superior parietal lobule; *lv*PREC = left ventral precuneus; rIPS = right intraparietal sulcus.
**Figure S8:**. Modulatory effects of each task condition (B matrix). We report the connections that exceeded the 95% posterior probability threshold in the PEB analysis. Warm colours in the extrinsic connections indicate more excitation as a result of the task condition, while cold colours signal more inhibition as an effect of task. In the case of self‐connections, the modulation is interpreted as increased (warm colours) or decreased (cold colours) inhibition, which means self‐connections become less or more sensitive (respectively) to inputs from the rest of the network as a result of task influence. Abbreviations: *ld*PREC = left dorsal precuneus; *l*FEF = left frontal eye fields; *li*OFC = left inferior orbitofrontal cortex; *l*IPL = left inferior parietal lobule; *lm*PFC = left middle prefrontal cortex; *l*SPL = left superior parietal lobule; *lv*PREC = left ventral precuneus; RE = external repetitions; RI = internal repetitions; rIPS = right intraparietal sulcus; SWE = switches to external; WI = switches to internal.
**Figure S9:**. Driving inputs from the task (C matrix), representing where each task condition enters the network. We report the connections that exceeded the 95% posterior probability threshold in the PEB analysis. Warm colours indicate a positive drive from task condition while cold colours indicate a negative drive. By definition, driving inputs are only present in self‐connections. Abbreviations: *ld*PREC = left dorsal precuneus; *l*FEF = left frontal eye fields; *li*OFC = left inferior orbitofrontal cortex; *l*IPL = left inferior parietal lobule; *lm*PFC = left middle prefrontal cortex; *l*SPL = left superior parietal lobule; *lv*PREC = left ventral precuneus; RE = external repetitions; RI = internal repetitions; rIPS = right intraparietal sulcus; SWE = switches to external; SWI = switches to internal.

## Data Availability

The data that support the findings of this study are available from the corresponding author upon reasonable request.
