# Supplementary material for: Neural Correlates of Goal‐Directed Preparation to Switching Across External and Internal Domains
Source: Hum Brain Mapp. 2025 Oct 15;46(15):e70376. doi: 10.1002/hbm.70376 (PMC12522180; doi:10.1002/hbm.70376)
Supplement: Supplementary file 1 — Table S1: List of all GLM pairwise contrasts. Table S2: One sample t tests over switch types after subtracting repetitions to check that all switch types in the 2 × 2 rANOVA were significantly different from task repetitions (=0). Table S3:. Descriptive statistics (mean and SD) of RTs for all conditions in the 2 × 2 rANOVA (domain × switch type). Table S4:. Results from the second‐level GLM analyses (one‐sample t test) testing for the group effects of domain on brain activation. Specific contrasts include: internal repetitions vs. external repetitions, external repetitions versus internal repetitions, switches to internal versus switches to external, switches to external vs. switches to internal. Results survived a threshold of p < 0.05 with family wise error (FWE) correction. Abbreviations: RE = repetition of external tasks; RI = repetition of internal tasks; SE = all switches towards external tasks; SI = all switches towards internal tasks. Table S5: Results from the second‐level GLM analyses (one‐sample t test) testing for the group effects of task repetition vs. task switching on brain activation. Specific contrasts include: internal repetitions versus switches to internal, external repetitions vs. switches to external. Results survived a threshold of p < 0.05 with family wise error (FWE) correction. Abbreviations: RE = repetition of external tasks; RI = repetition of internal tasks; SE = all switches towards external tasks; SI = all switches towards internal tasks. Table S6: fMRI results on the effects of domain—additional regressor analysis. Results from the second‐level GLM analyses (one‐sample t test) testing for the group effects of domain on brain activation, including an additional regressor of no interest (between stimulus onset and button press) to further control for the effect of motor preparation. Specific contrasts include: internal versus external domain (all trial types included), external versus internal domain (all trial types included). Results [file HBM-46-e70376-s001.docx]

**Supplementary Materials**

| **GLM contrasts** | | | | | |
| --- | --- | --- | --- | --- | --- |
|  |  | **Condition A > Condition B** | | | **Conjunction** |
| **Effect of Domain** | **1** | Internal (all trial types) | > | External (all trial types) |  |
|  | **2** | External (all trial types) | > | Internal (all trial types) |  |
| **Effect of Switching** | **3** | Switches (both domains) | > | Repetitions (both domains) |  |
|  | **4** | Switches to internal | > | Repetitions of internal | X |
|  | **5** | Switches to external | > | Repetitions of external |  |
|  | **6** | Switches within internal | > | Repetitions of internal | X |
|  | **7** | Switches within external | > | Repetitions of external |  |
|  | **8** | External-to-internal switches | > | Repetitions of internal | X |
|  | **9** | Internal-to-external switches | > | Repetitions of external |  |
| **Comparison across switch types** | **10** | All between-domain switches | > | All within-domain switches |  |
|  | **11** | External-to-internal switches | > | Switches within internal | X |
|  | **12** | Internal-to-external switches | > | Switches within external |  |

**Table S1.** List of all GLM pairwise contrasts.

|  |  | **frequentist** | | | | **Bayesian** |
| --- | --- | --- | --- | --- | --- | --- |
|  |  | **t** | **df** | **p** | **Cohen’s d** | **BF_10_** |
|  | **within internal** | 4.675 | 29 | < .001 | 0.854 | 396.799 |
|  | **between internal** | 5.747 |  | < .001 | 1.049 | 6035.418 |
|  | **within external** | 6.918 |  | < .001 | 1.263 | 115045.595 |
|  | **between external** | 6.914 |  | < .001 | 1.262 | 113990.771 |

**Table S2.** One sample t-tests over switch types after subtracting repetitions to check that all switch types in the 2x2 rANOVA were significantly different from task repetitions (=0).

|  | **Repetitions** | **Combined switches** | **Within-domain sw.** | **Between-domain sw.** |
| --- | --- | --- | --- | --- |
| **Internal** | 1.161 (0.220) | 1.252 (0.235) | 1.231 (0.256) | 1.274 (0.223) |
| **External** | 1.099 (0.168) | 1.250 (0.205) | 1.224 (0.207) | 1.272 (0.230) |

**Table S3.** Descriptive statistics (mean and SD) of RTs for all conditions in the 2x2 rANOVA (domain x switch type).

| Contrast | Region (AAL3) | Peak Coordinates | Voxels per cluster | z-score | Peak p_FWE-corr_ | Cluster p_FWE-corr_ |
| --- | --- | --- | --- | --- | --- | --- |
| RI > RE | L Inferior Temporal | -47 5.5 -35 | 238 | 6.859 | <.001 | .000 |
|  | R Crus I (Uvula) | 30.5 -84.5 -32.5 | 383 | 6.841 | <.001 | .000 |
|  | L Inferior Orbitofrontal | -49.5 30.5 -5 | 327 | 6.411 | <.001 | .000 |
|  | L Medial Superior Frontal | -4.5 50.5 22.5 | 374 | 6.377 | <.001 | .000 |
|  | L Middle Temporal | -54.5 -34.5 0 | 35 | 6.053 | <.001 | <.001 |
|  | L Superior Frontal | -12 38 50 | 58 | 6.005 | <.001 | <.001 |
|  | Undefined | -9.5 0.5 7.5 | 44 | 5.960 | <.001 | <.001 |
|  | L Mid / Post Cingulate | -9.5 -42 35 | 61 | 5.945 | <.001 | <.001 |
|  | L Crus II (Uvula) | -29.5 -84.5 -35 | 20 | 5.813 | <.001 | <.001 |
|  | L Inf Frontal, pars triangularis | -52 20.5 22.5 | 12 | 5.545 | 0.001 | <.001 |
|  | L Rectus | -2 45.5 -20 | 24 | 5.469 | 0.002 | <.001 |
|  | L Middle Cingulate | -4.5 -12 37.5 | 7 | 5.461 | 0.002 | <.001 |
|  | L Lingual / Parahippocampal | -9.5 -42 0 | 5 | 5.377 | 0.004 | <.001 |
|  | Undefined | -12 -24.5 -7.5 | 4 | 5.342 | 0.004 | 0.001 |
|  | L Supramarginal / Mid Temporal | -52 -54.5 22.5 | 6 | 5.341 | 0.004 | <.001 |
|  | L Superior Occipital / Cuneus | -12 -99.5 17.5 | 7 | 5.329 | 0.005 | <.001 |
|  | R Cerebellar Tonsil | 5.5 -52 -45 | 3 | 5.326 | 0.005 | 0.003 |
|  | R Inf Frontal, pars triangularis | 55.5 30.5 -2.5 | 12 | 5.256 | 0.007 | <.001 |
|  | L Amygdala / Parahippocampal | -24.5 -7 -15 | 7 | 5.225 | 0.008 | <.001 |
|  | L Cuneus | -2 -77 32.5 | 6 | 5.214 | 0.009 | <.001 |
|  | L Superior Frontal | -19.5 33 42.5 | 3 | 5.170 | 0.011 | 0.003 |
|  | R Mid Temporal Pole / STG | 40.5 13 -37.5 | 4 | 5.140 | 0.013 | 0.001 |
|  | R SMA | 8 18 62.5 | 3 | 5.064 | 0.020 | 0.003 |
|  | L Amygdala / Parahippocampal | -29.5 -4.5 -20 | 3 | 5.034 | 0.023 | 0.003 |
|  | L Superior Frontal | -19.5 25.5 45 | 4 | 5.032 | 0.023 | 0.001 |
| RE > RI | R Superior Parietal / Precuneus | 18 -64.5 55 | 635 | 6.615 | <.001 | 0.000 |
|  | L Inferior Parietal | -34.5 -42 45 | 671 | 6.523 | <.001 | 0.000 |
|  | L Superior Frontal | -24.5 -4.5 50 | 36 | 5.846 | <.001 | <.001 |
|  | R Superior Frontal | 33 0.5 60 | 13 | 5.379 | 0.004 | <.001 |
|  | L Inf Semi-Lunar Lobule | -29.5 -69.5 -52.5 | 4 | 5.160 | 0.012 | 0.001 |
| SI > SE | L Precuneus | -4.5 -52 7.5 | 278 | 6.571 | <.001 | 0.000 |
|  | L Inf Frontal, pars Orbitalis | -49.5 30.5 -5 | 375 | 6.494 | <.001 | 0.000 |
|  | L Angular | -49.5 -57 25 | 103 | 6.403 | <.001 | <000 |
|  | R Superior Temporal Pole | 45.5 20.5 -30 | 11 | 6.240 | <.001 | <.001 |
|  | L Rectus | -2 33 -17.5 | 21 | 6.102 | <.001 | <.001 |
|  | L SMA | -4.5 20.5 62.5 | 25 | 5.977 | <.001 | <.001 |
|  | L Middle Temporal | -52 -37 0 | 19 | 5.866 | <.001 | <.001 |
|  | L Superior Frontal | -12 50.5 30 | 59 | 5.851 | <.001 | <.001 |
|  | L Superior Frontal | -19.5 33 45 | 22 | 5.739 | <.001 | <.001 |
|  | L pre-Anterior Cingulate | -9.5 43 12.5 | 9 | 5.643 | <.001 | <.001 |
|  | L Middle Temporal Pole | -47 13 -35 | 142 | 5.594 | 0.001 | 0.000 |
|  | R Crus II | 15.5 -87 -32.5 | 25 | 5.504 | 0.002 | <.001 |
|  | R Middle Temporal | 58 -4.5 -20 | 8 | 5.360 | 0.004 | <.001 |
|  | L Cuneus | -12 -925 25 | 5 | 5.262 | 0.007 | <.001 |
|  | L Mid Frontal | -34.5 18 50 | 3 | 5.120 | 0.014 | 0.002 |
|  | L Inf Orbitofrontal | -32 33 -7.5 | 4 | 5.022 | 0.024 | 0.001 |
| SE > SI | L Superior Frontal | -24.5 -4.5 50 | 58 | 6.478 | <.001 | <.001 |
|  | L Mid Occipital | -27 -64.5 32.5 | 199 | 6.147 | <.001 | 0.000 |
|  | L Inferior Parietal | -34.5 -39.5 42.5 | 59 | 5.895 | <.001 | <.001 |
|  | R Middle Frontal | 25.5 0.5 50 | 41 | 5.836 | <.001 | <.001 |
|  | R Supramarginal / IPL | 45.5 -34.5 42.5 | 22 | 5.716 | <.001 | <.001 |
|  | R Angular/ Sup Par Lobule | 25.5 -62 47.5 | 39 | 5.433 | 0.003 | <.001 |

**Table S4.** Results from the second-level GLM analyses (one-sample t-test) testing for the group effects of domain on brain activation. Specific contrasts include: internal repetitions vs external repetitions, external repetitions vs internal repetitions, switches to internal vs switches to external, switches to external vs switches to internal. Results survived a threshold of p < 0.05 with family wise error (FWE) correction. Abbreviations: RI = repetition of internal tasks; RE = repetition of external tasks; SI = all switches towards internal tasks; SE = all switches towards external tasks.

| Contrast | Region (AAL3) | Peak Coordinates | Voxels per cluster | z-score | Peak p_FWE-corr_ | Cluster p_FWE-corr_ |
| --- | --- | --- | --- | --- | --- | --- |
| RI > SI | R Crus I (Uvula) | 28 -79.5 -32.5 | 30 | 5.441 | <.001 | .003 |
|  | **L Medial Superior Frontal** | -12 48 20 | 26 | 5.773 | <.001 | <.001 |
|  | **L Medial Superior Frontal** | 0.5 50.5 37.5 | 10 | 5.665 | <.001 | <.001 |
| RE > SE | No voxel survived FWE correction | |  |  |  |  |
|  |  |  |  |  |  |  |

**Table S5.** Results from the second-level GLM analyses (one-sample t-test) testing for the group effects of task repetition vs task switching on brain activation. Specific contrasts include: internal repetitions vs switches to internal, external repetitions vs switches to external. Results survived a threshold of p < 0.05 with family wise error (FWE) correction. Abbreviations: RI = repetition of internal tasks; SI = all switches towards internal tasks; RE = repetition of external tasks; SE = all switches towards external tasks.

| Contrast | Region (AAL3) | Peak Coordinates | Voxels per cluster | z-score | Peak p_FWE-corr_ | Cluster p_FWE-corr_ |
| --- | --- | --- | --- | --- | --- | --- |
| 1. I > E | L Precuneus | -4.5 -52 12.5 | 38 | 5.891 | <0.001 | <0.001 |
|  | L Inferior Orbitofrontal | -44.5 28 -12.5 | 198 | 5.825 | <0.001 | 0.000 |
|  | R Crus II | 30.5 -72 -40 | 49 | 5.801 | <0.001 | <0.001 |
|  | L Medial Superior Frontal | -4.5 45.5 40 | 16 | 5.773 | <0.001 | <0.001 |
|  | L Angular | -47 -64.5 30 | 53 | 5.575 | 0.001 | <0.001 |
|  | L Superior Occipital | -2 -99.5 15 | 11 | 5.452 | 0.002 | <0.001 |
|  | L Rectus (Medial Frontal) | -2 40.5 -17.5 | 24 | 5.442 | 0.002 | <0.001 |
|  | L Cuneus | -2 -69.5 30 | 13 | 5.423 | 0.003 | <0.001 |
|  | L Mid Frontal | -34.5 18 52.5 | 4 | 5.415 | 0.003 | 0.001 |
|  | L Posterior Cingulate | -2 –47 25 | 17 | 5.369 | 0.004 | <0.001 |
|  | L Middle Cingulate | -9.5 -42 35 | 11 | 5.367 | 0.004 | <0.001 |
|  | R Crus II | 13 –89.5 -30 | 8 | 5.314 | 0.005 | <0.001 |
|  | L Supplementary Motor Area | -4.5 20.5 62.5 | 3 | 5.246 | 0.007 | 0.002 |
|  | L Inf Frontal, pars triangularis | -52 20.5 22.5 | 4 | 5.171 | 0.011 | 0.001 |
|  |  |  |  |  |  |  |
| 1. E > I | L Superior Frontal (FEF) | -24.5 -4.5 50 | 47 | 6.332 | <0.001 | <0.001 |
|  | L Inferior Parietal | -34.5 -39.5 42.5 | 53 | 5.907 | <0.001 | <0.001 |
|  | L Superior Parietal | -27 -52 55 | 36 | 5.686 | <0.001 | <0.001 |
|  | R Supramarginal | 45.5 -34.5 40 | 8 | 5.500 | 0.002 | <0.001 |
|  | undefined | -24.5 -64.5 30 | 9 | 5.396 | 0.003 | <0.001 |
|  | R Superior Parietal | 25.5 -62 50 | 6 | 5.136 | 0.013 | <0.001 |

**Table S6.** **fMRI results on the effects of domain – additional regressor analysis.** Results from the second-level GLM analyses (one-sample t-test) testing for the group effects of domain on brain activation, including an additional regressor of no interest (between stimulus onset and button press) to further control for the effect of motor preparation. Specific contrasts include: internal vs external domain (all trial types included), external vs internal domain (all trial types included). Results survived a threshold of p < 0.05 with family wise error (FWE) correction. Abbreviations: I = internal tasks; E = external tasks.

| Contrast | Region (AAL3) | Peak Coordinates | Voxels per cluster | z-score | Peak p_FWE-corr_ | Cluster p_FWE-corr_ |
| --- | --- | --- | --- | --- | --- | --- |
| S > R | L Precuneus | -9.5 -69.5 47.5 | 81 | 6.238 | <0.001 | <0.001 |
|  | L Precentral / Inferior Frontal | -47 3 32.5 | 10 | 5.680 | <0.001 | <0.001 |
|  | L Inferior Parietal | -27 –59.5 42.5 | 19 | 5.486 | 0.002 | <0.001 |
|  | L Supplementary Motor Area | -9.5 3 62.5 | 4 | 5.425 | 0.003 | 0.001 |
|  | L Superior Frontal | -24.5 0.5 55 | 11 | 5.420 | 0.003 | <0.001 |
|  | R Inferior Semi-Lunar Lobule | 30.5 -69.5 -52.5 | 9 | 5.336 | 0.004 | <0.001 |
|  | L Inferior Parietal | -39.5 -49.5 42.5 | 30 | 5.139 | 0.013 | <0.001 |
| SI > RI | L Precuneus | -7 -69.5 52.5 | 35 | 5.820 | <0.001 | <0.001 |
|  | L Superior Parietal Lobule | -29.5 -59.5 45 | 12 | 5.376 | 0.004 | <0.001 |
|  | R Superior Parietal / Precuneus | 13 -64.5 55 | 15 | 5.345 | 0.004 | <0.001 |
|  | L Superior Parietal Lobule | -24.5 -64.5 57.5 | 8 | 5.237 | 0.008 | <0.001 |
|  | L Inferior Parietal Lobule | -49.5 -34.5 45 | 7 | 5.100 | 0.016 | <0.001 |
|  | L Precuneus | -9.5 -67 40 | 5 | 5.011 | 0.026 | <0.001 |
|  | L Postcentral | -42 –39.5 60 | 3 | 5.002 | 0.027 | 0.002 |
| SE > RE | R Crus II | 25.5 -84.5 -37.5 | 9 | 5.108 | 0.015 | <0.001 |
|  | L Superior Frontal | -24.5 -4.5 50 | 3 | 4.937 | 0.038 | 0.002 |
| SwI > RI | L Precuneus | -7 –69.5 52.5 | 35 | 5.820 | <0.001 | <0.001 |
|  | L Superior Parietal Lobule | -29.5 -59.5 45 | 12 | 5.376 | 0.004 | <0.001 |
|  | R Superior Parietal | 13 –64.5 55 | 15 | 5.345 | 0.004 | <0.001 |
|  | L Superior Parietal Lobule | -24.5 -64.5 57.5 | 8 | 5.237 | 0.008 | <0.001 |
|  | L Inferior Parietal Lobule | -49.5 -34.5 45 | 7 | 5.100 | 0.016 | <0.001 |
|  | L Precuneus | -9.5 -67 40 | 5 | 5.011 | 0.026 | <0.001 |
|  | L Postcentral | -42 –39.5 60 | 3 | 5.002 | 0.027 | 0.002 |
| SwE > RE | R Crus II | 25.5 -84.5 -37.5 | 9 | 5.109 | 0.015 | <0.001 |
|  | L Middle Frontal | -24.5 -4.5 50 | 3 | 4.937 | 0.038 | 0.002 |
| SbI > RI | L Postcentral | -42 –39.5 57.5 | 8 | 5.560 | 0.001 | <0.001 |
|  | L Preuneus | -9.5 -64.5 55 | 53 | 5.540 | 0.001 | <0.001 |
|  | L Superior Parietal Lobule | -24.5 -64.5 57.5 | 9 | 5.442 | 0.003 | <0.001 |
|  | R Superior Parietal | 15.5 -67 50 | 11 | 5.250 | 0.007 | <0.001 |
|  | L Inferior Parietal | -29.5 -59.5 42.5 | 4 | 5.150 | 0.012 | 0.001 |
|  | R Angular | 30.5 -67 45 | 5 | 5.127 | 0.014 | <0.001 |
|  | L Inferior Parietal Lobule | -49.5 -34.5 45 | 3 | 5.089 | 0.017 | 0.002 |
| SbE > RE | R Crus II | 23 –84.5 -40 | 169 | 5.987 | <0.001 | 0.000 |
|  | L Putamen | -17 8 0 | 6 | 5.297 | 0.006 | <0.001 |
|  | L Mid Cingulate | -4.5 20.5 37.5 | 9 | 5.268 | 0.007 | <0.001 |
|  | R Putamen | 15.5 13 -5 | 4 | 5.166 | 0.011 | 0.001 |
|  | L Ventral Anterior Thalamus | -7 –2 7.5 | 3 | 5.035 | 0.023 | 0.002 |

**Table S7.** **fMRI results on the effects of switching and differences between switch types – additional regressor analysis.** Results from the second-level GLM analyses (one-sample t-test) testing for the group effects of switching on brain activation, including an additional regressor of no interest (between stimulus onset and button press) to further control for the effect of motor preparation. Contrasts include: all switches vs all repetitions; switches to internal vs internal repetitions and switches to external vs external repetitions; switches within internal vs internal repetitions and switches within external vs external repetitions; external to internal switches vs internal repetitions and internal-external switches vs external repetitions. Results survived a threshold of p < 0.05 with family wise error (FWE) correction. Abbreviations: R = all repetitions; RI = repetition of internal tasks; RE = repetition of external tasks; S = all switches; SI = all switches towards internal tasks; SE = all switches towards external tasks; S_w_I = switches within internal tasks; S_w_E = switches within external tasks; S_b_I = switches from external to internal tasks (between-domain); S_b_E = switches from internal to external tasks (between-domain).

| Contrast | Region (AAL3) | Peak Coordinates | Voxels per cluster | z-score | Peak p_FWE-corr_ | Cluster p_FWE-corr_ |
| --- | --- | --- | --- | --- | --- | --- |
| SbI > SwI | No voxels survived FWE correction | |  |  |  |  |
| SbE > SwE | R Crus II | 18 –89.5 -37.5 | 127 | 6.180 | <0.001 | 0.000 |
|  | L Superior Medial Frontal | -9.5 33 35 | 3 | 5.404 | 0.003 | 0.002 |

**Table S8.** **fMRI results on the differences between switch types – additional regressor analysis.** Results from the second-level GLM analyses (one-sample t-test) testing for the group differences between switch types, including an additional regressor of no interest (between stimulus onset and button press) to further control for the effect of motor preparation. Contrasts include: switches from external to internal vs. switches within the internal domain; switches from internal to external vs. switches within the external domain. Results survived a threshold of p < 0.05 with family wise error (FWE) correction. Abbreviations: S_w_I = switches within internal tasks; S_w_E = switches within external tasks; S_b_I = switches from external to internal tasks (between-domain); S_b_E = switches from internal to external tasks (between-domain).

| Region (AAL3) | Peak Coordinates |
| --- | --- |
| L Inferior Orbitofrontal | -49.5 30.5 -5 |
| L ventral Precuneus | -4.5 -49.5 10 |
| L Medial Superior Frontal | -4.5 45.5 37.5 |
| L Superior Parietal | -27 -54.5 55 |
| R Supramarginal | 45.5 -34.5 42.5 |
| L Superior Frontal | -24.5 -4.5 50 |
| L Inferior Parietal Lobule | -37 -42 40 |
| L dorsal Precuneus | -9.5 -69.5 47.5 |

**Table S9. Difference in brain activation between domains.** These coordinates were extracted from GLM T-contrasts and formed the starting point for the search of individual coordinates in the GLM activation maps. Orbitofrontal, ventral precuneus and medial superior frontal cortex were extracted from the contrast ‘internal > external’; superior parietal, supramarginal and lateral superior frontal were extracted from ‘external > internal’; finally, IPL and dorsal precuneus were extracted from ‘switches > repetitions’ (all spheres of 6 mm radius).


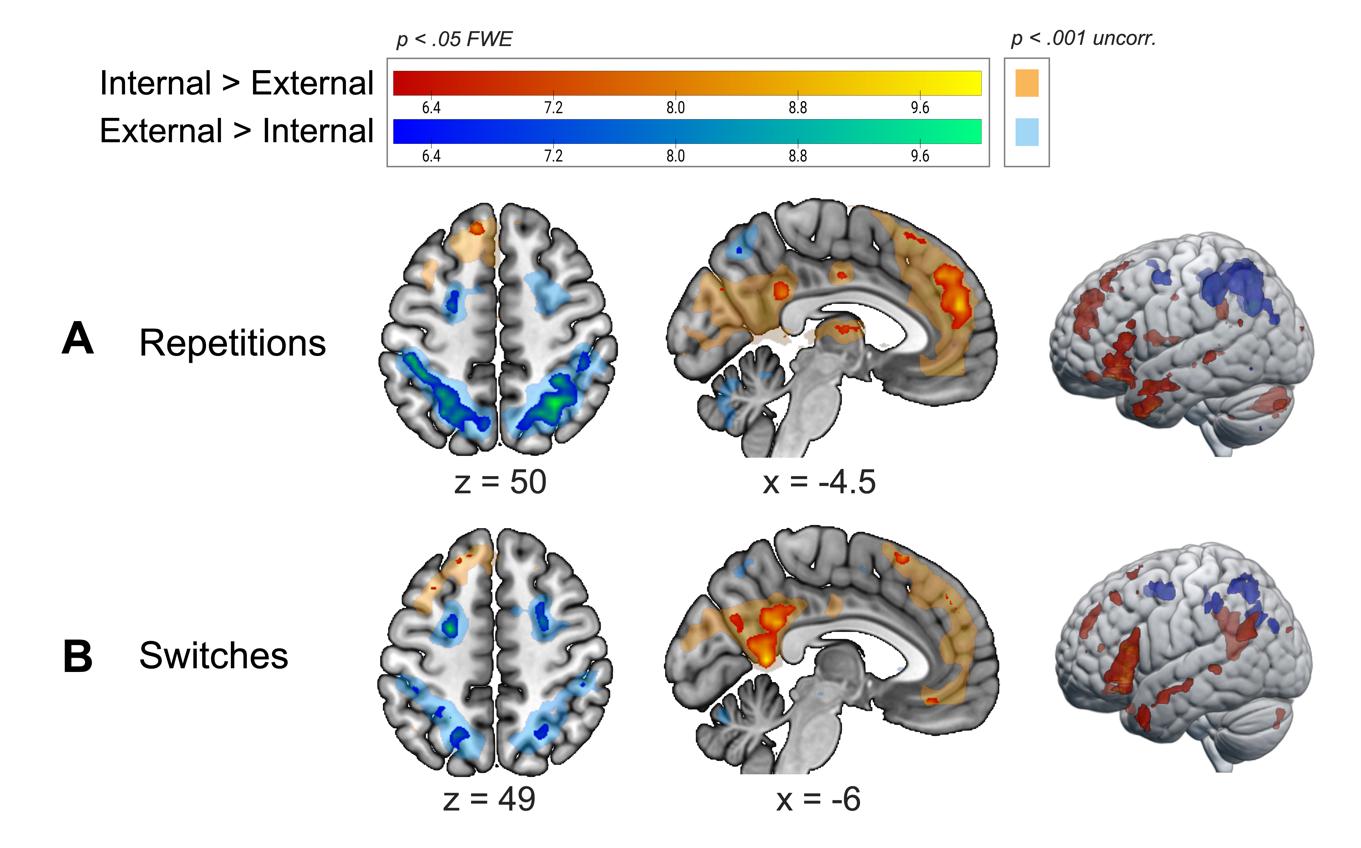


**Figure S1.** Brain activation at the group level (one-sample t-test) for the whole-brain comparisons between domains for each trial type. Specifically, the first-level GLMs compared ‘internal > external’ and ‘external > internal’ for both repetitions only (A) and switches only (B) on a multi-run model for each subject (4 runs of task per participant). The activation maps are shown at p<0.05 FWE-corrected (bright colours; warm scale = internal > external; cold scale = external > internal) and rendered on a standard template (mni152 template in MRIcroGL). Uncorrected activation maps at *p*<.001 are also shown for representational purposes with faint, transparent colours. For each slice, the relative MNI coordinate is reported.


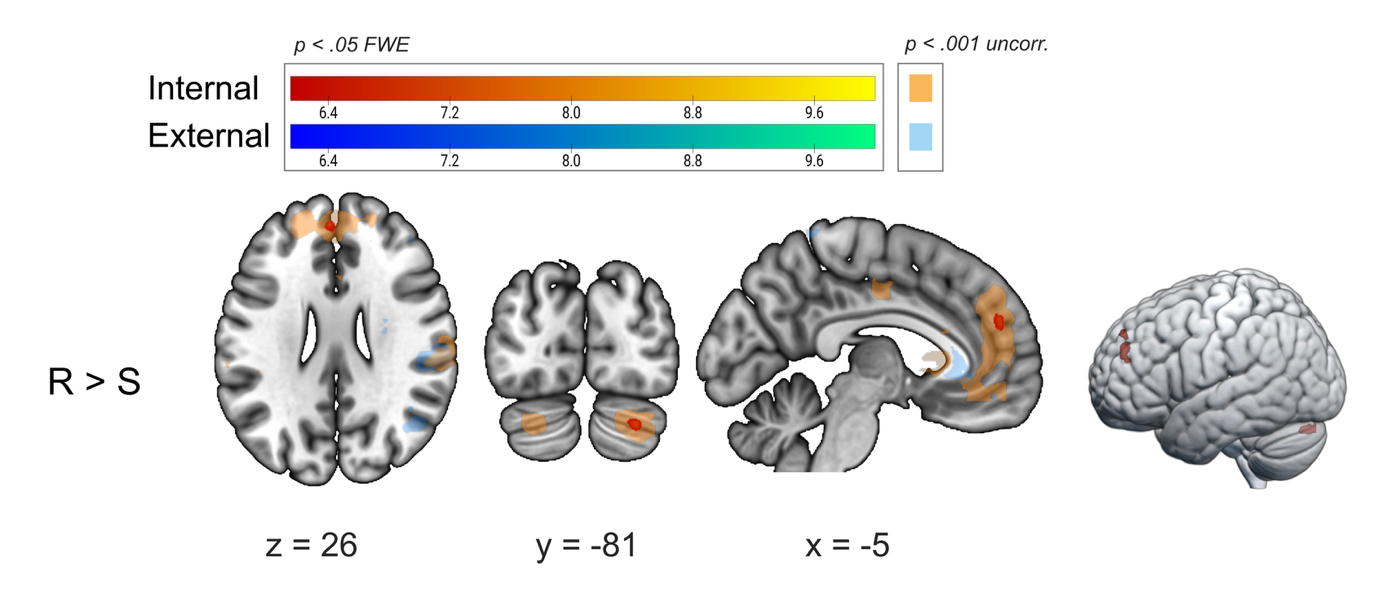


**Figure S2.** Brain activation at the group level (one-sample t-test) for the whole-brain comparisons between repetitions and switches for each domain type. Specifically, the first-level GLMs compared ‘internal repetitions > switches to internal’ and ‘external repetitions > switches to external’ on a multi-run model for each subject (4 runs of task per participant). The activation maps are shown at p<0.05 FWE-corrected (bright colours; warm scale = internal domain; cold scale = external domain) and rendered on a standard template (mni152 template in MRIcroGL). Uncorrected activation maps at *p*<.001 are also shown for representational purposes with faint, transparent colours. For each slice, the relative MNI coordinate is reported.


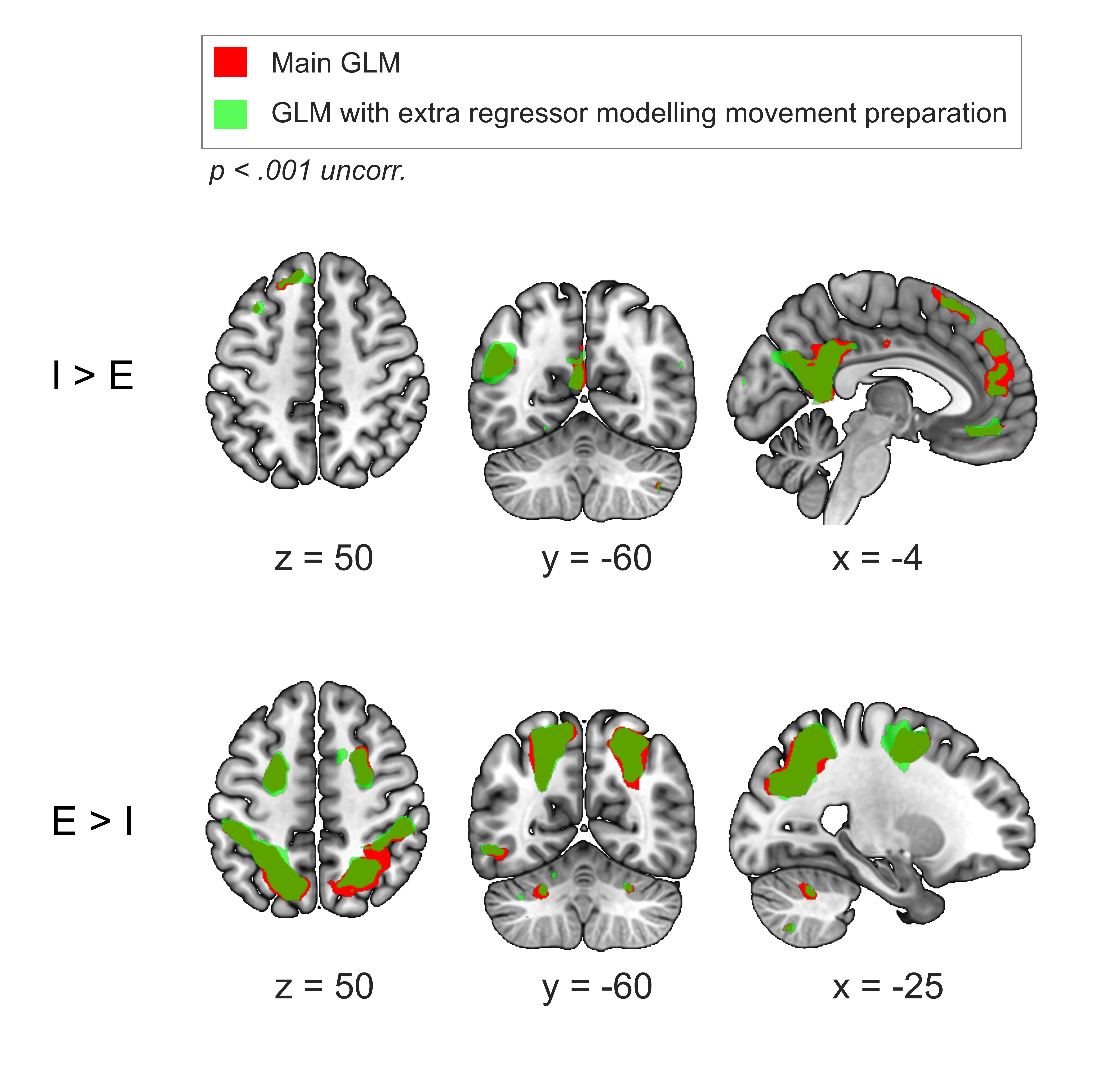


**Figure S3.** **Difference in brain activation between GLM versions – Effect of domain.** Activation at the group level (one-sample t-test) for the whole-brain comparisons between domains, regardless of trial type. In both versions, the first-level GLMs compared ‘internal > external’ and ‘external > internal’ on a multi-run model for each subject (4 runs of task per participant). The main GLM (red) only included the button response as regressor of no interest, while the alternative GLM (green) also included a regressor for motor preparation (period between stimulus onset and button press). The uncorrected activation maps are shown at p<0.001 and rendered on a standard template (mni152 template in MRIcroGL). For each slice, the relative MNI coordinate is reported.


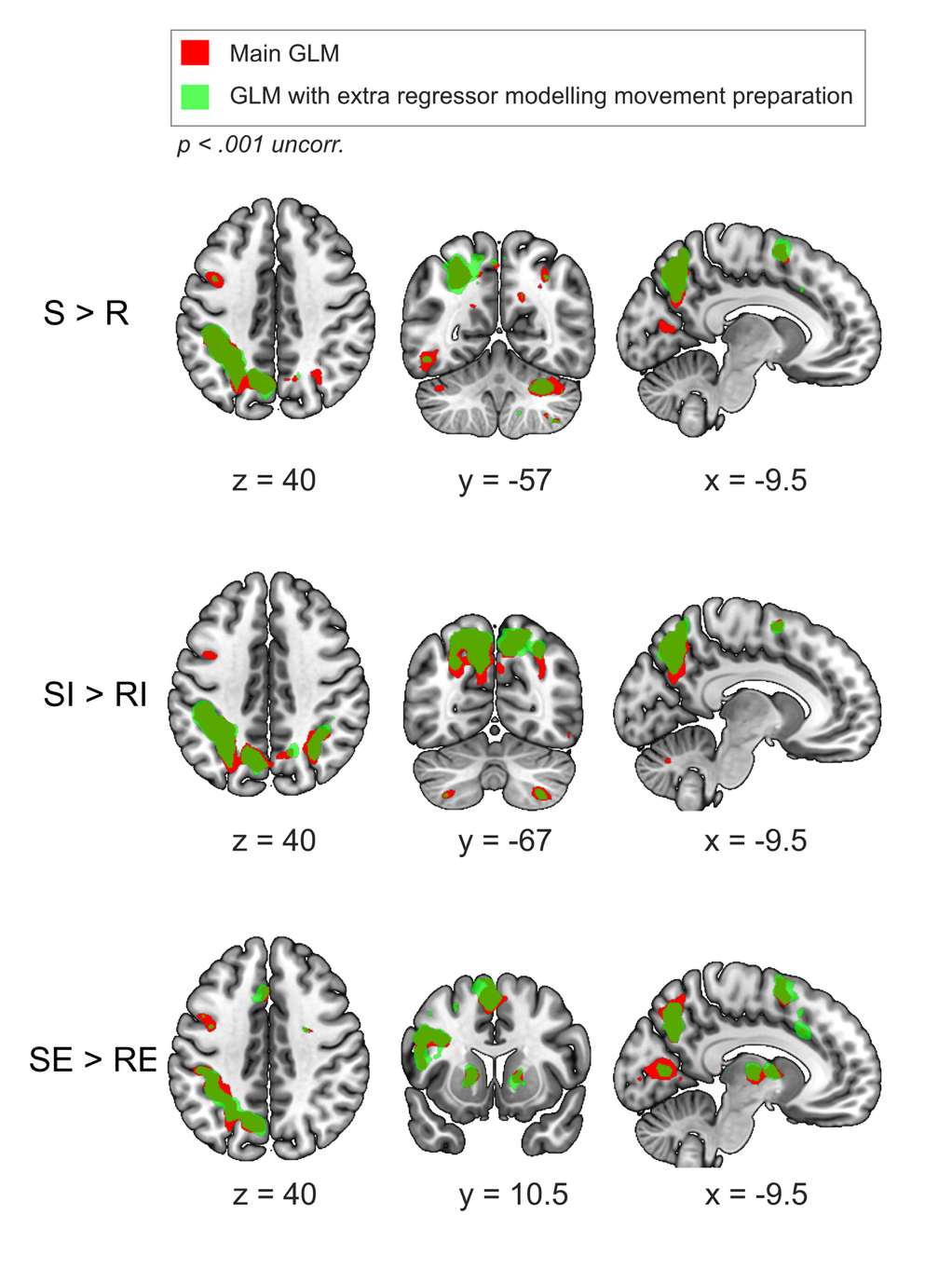


**Figure S4.** **Difference in brain activation between GLM versions – Effect of switches vs repetitions.** Activation at the group level (one-sample t-test) for the whole-brain comparisons between switches and repetitions. In both versions, the first-level GLMs compared ‘all switches > all repetitions’, ‘internal switches > internal repetitions’ and ‘external switches > external repetitions’ on a multi-run model for each subject (4 runs of task per participant). The main GLM (red) only included the button response as regressor of no interest, while the alternative GLM (green) also included a regressor for motor preparation (period between stimulus onset and button press). The uncorrected activation maps are shown at p<0.001 and rendered on a standard template (mni152 template in MRIcroGL). For each slice, the relative MNI coordinate is reported.


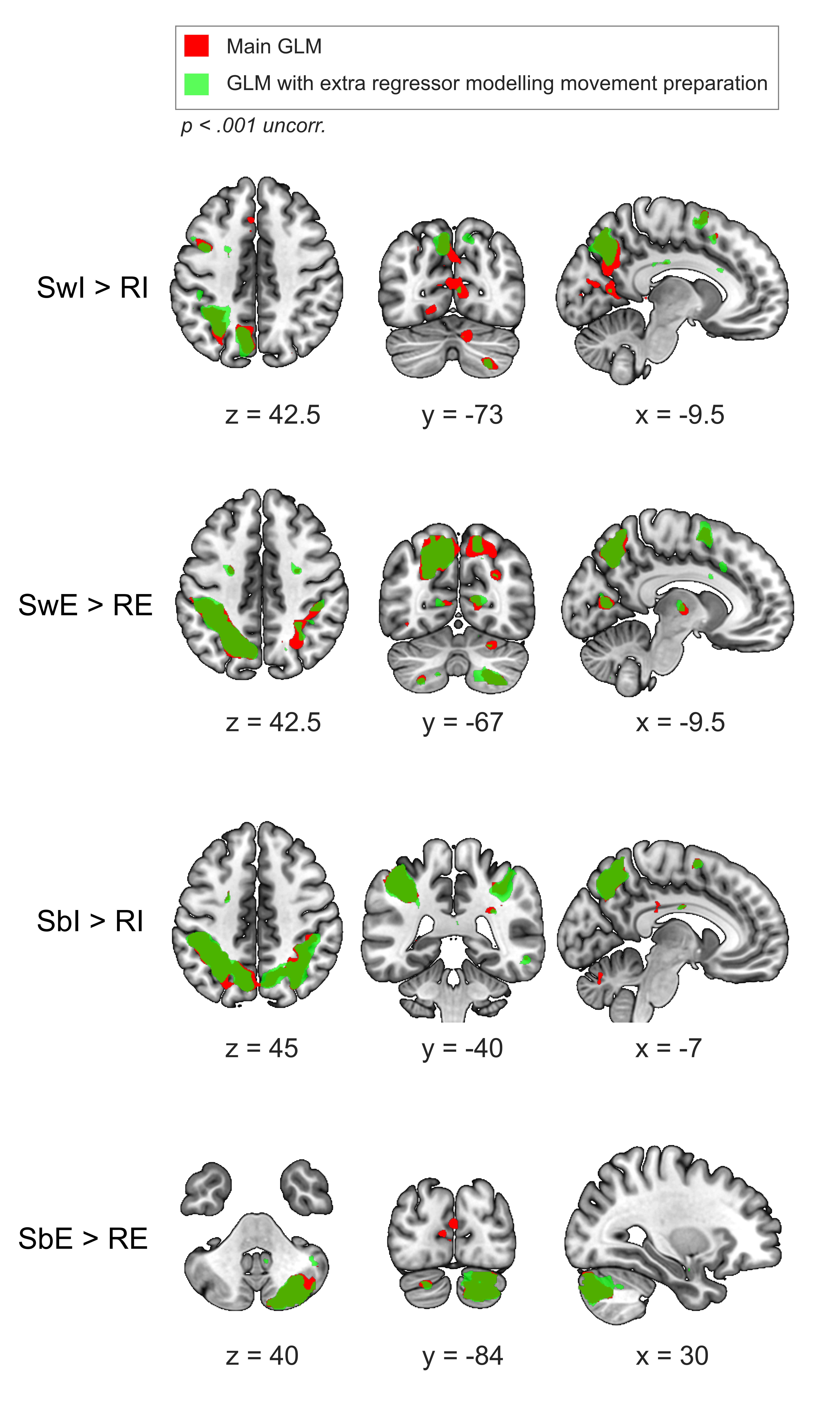


**Figure S5.** **Difference in brain activation between GLM versions – Effect of specific switch types vs repetitions.** Activation at the group level (one-sample t-test) for the whole-brain comparisons between specific switch types and repetitions. In both versions, the first-level GLMs compared ‘switches within internal > internal repetitions’, ‘switches within external > external repetitions’, ‘switches from external to internal > internal repetitions’ and ‘switches from internal to external > external repetitions’ on a multi-run model for each subject (4 runs of task per participant). The main GLM (red) only included the button response as regressor of no interest, while the alternative GLM (green) also included a regressor for motor preparation (period between stimulus onset and button press). The uncorrected activation maps are shown at p<0.001 and rendered on a standard template (mni152 template in MRIcroGL). For each slice, the relative MNI coordinate is reported.


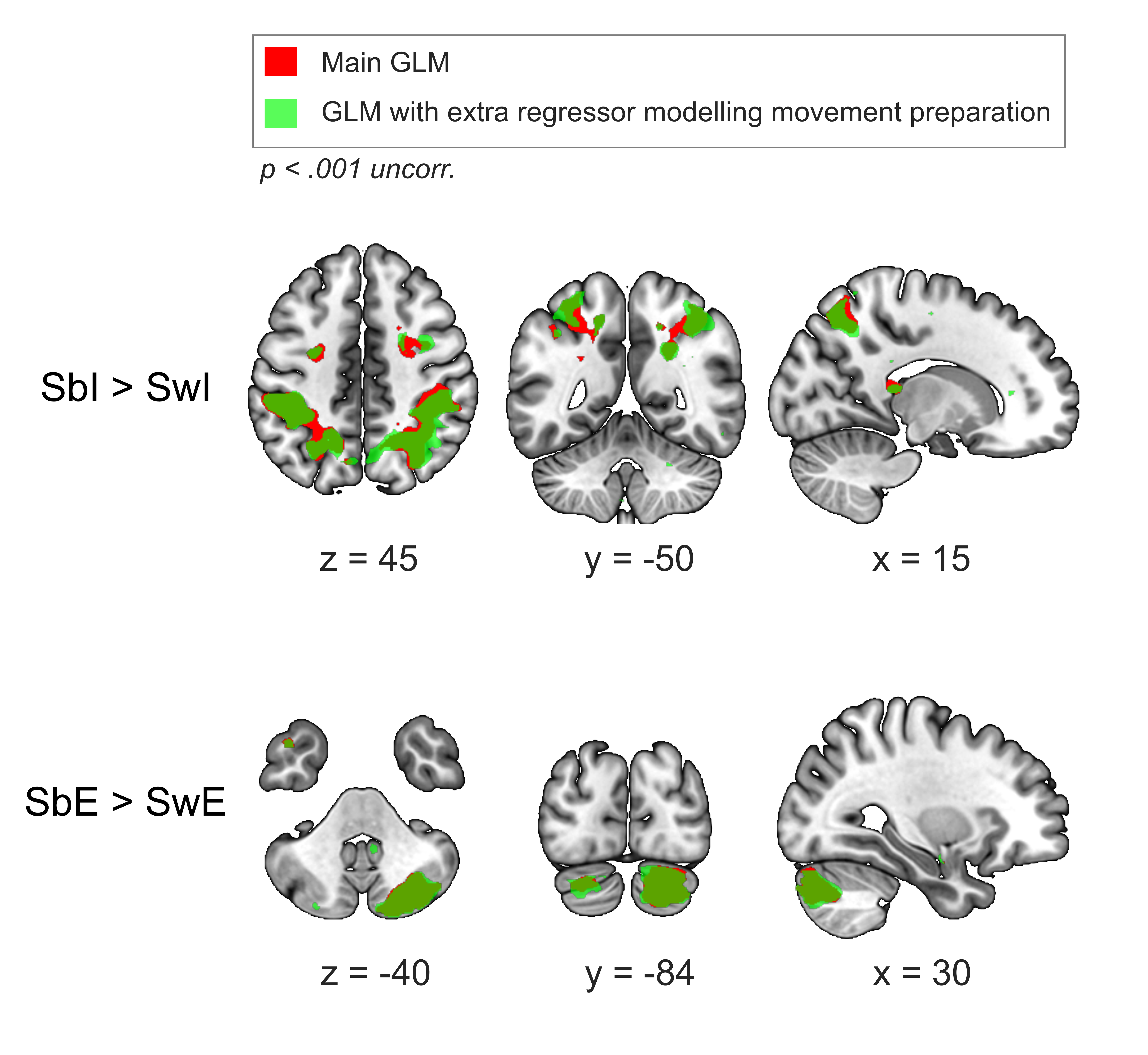


**Figure S6.** **Difference in brain activation between GLM versions – Comparison between switch types.** Activation at the group level (one-sample t-test) for the whole-brain comparisons between switch types. In both versions, the first-level GLMs compared ‘switches from external to internal > switches within internal’ and ‘switches from internal to external > switches within external’ on a multi-run model for each subject (4 runs of task per participant). The main GLM (red) only included the button response as regressor of no interest, while the alternative GLM (green) also included a regressor for motor preparation (period between stimulus onset and button press). The uncorrected activation maps are shown at p<0.001 and rendered on a standard template (mni152 template in MRIcroGL). For each slice, the relative MNI coordinate is reported.


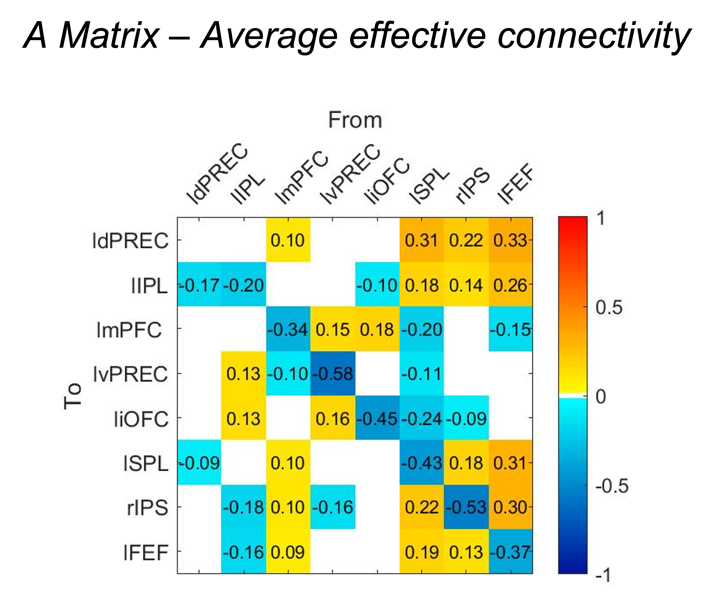


**Figure S7.** Estimated average effective connectivity across experimental conditions (A matrix). We report the connections that exceeded the 95% posterior probability threshold in the PEB analysis. Warm colours in the extrinsic connections indicate more excitation as compared to baseline (i.e., rest), cold colours signal more inhibition compared to baseline. Note that self-connections are log-scaled and always inhibitory, so warm colours indicate a reduction in inhibition and cold colours indicate increased inhibition. Abbreviations: *lm*PFC = left middle prefrontal cortex; *lv*PREC = left ventral precuneus; *li*OFC = left inferior orbitofrontal cortex; *l*SPL = left superior parietal lobule; rIPS = right intraparietal sulcus; *l*FEF = left frontal eye fields; *l*IPL = left inferior parietal lobule; *ld*PREC = left dorsal precuneus.


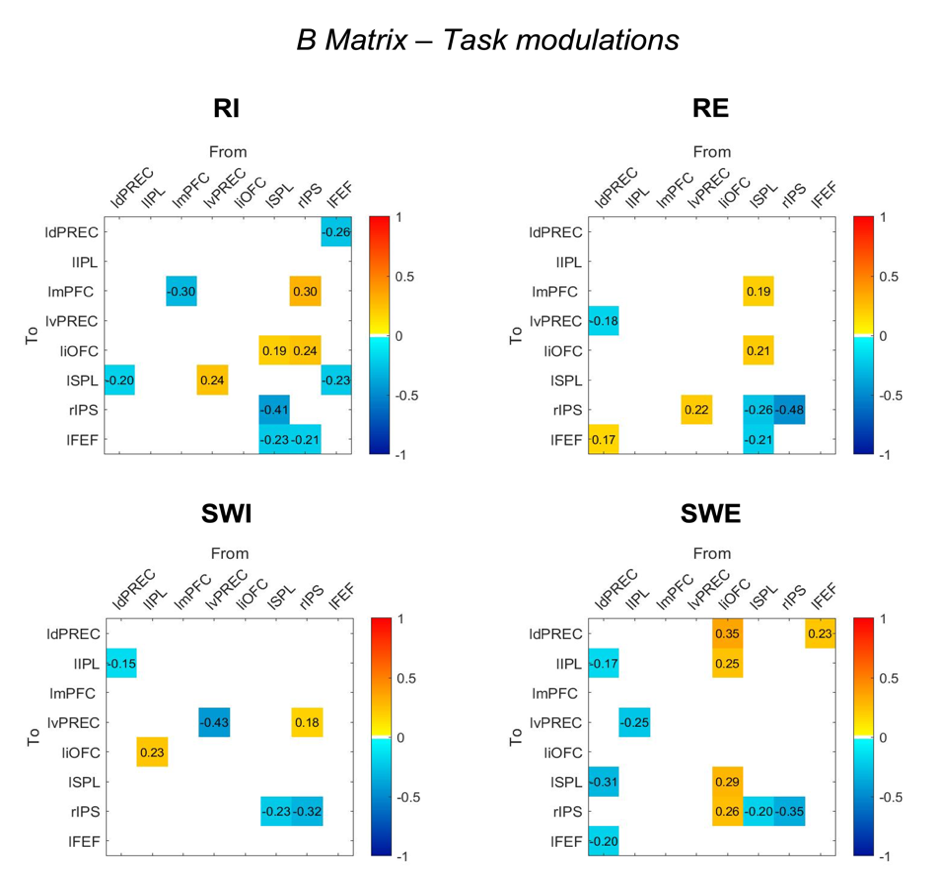


**Figure S8.** Modulatory effects of each task condition (B matrix). We report the connections that exceeded the 95% posterior probability threshold in the PEB analysis. Warm colours in the extrinsic connections indicate more excitation as a result of the task condition, while cold colours signal more inhibition as an effect of task. In the case of self-connections, the modulation is interpreted as increased (warm colours) or decreased (cold colours) inhibition, which means self-connections become less or more sensitive (respectively) to inputs from the rest of the network as a result of task influence. Abbreviations: RI = internal repetitions; RE = external repetitions; SWI = switches to internal; SWE = switches to external; *lm*PFC = left middle prefrontal cortex; *lv*PREC = left ventral precuneus; *li*OFC = left inferior orbitofrontal cortex; *l*SPL = left superior parietal lobule; rIPS = right intraparietal sulcus; *l*FEF = left frontal eye fields; *l*IPL = left inferior parietal lobule; *ld*PREC = left dorsal precuneus.


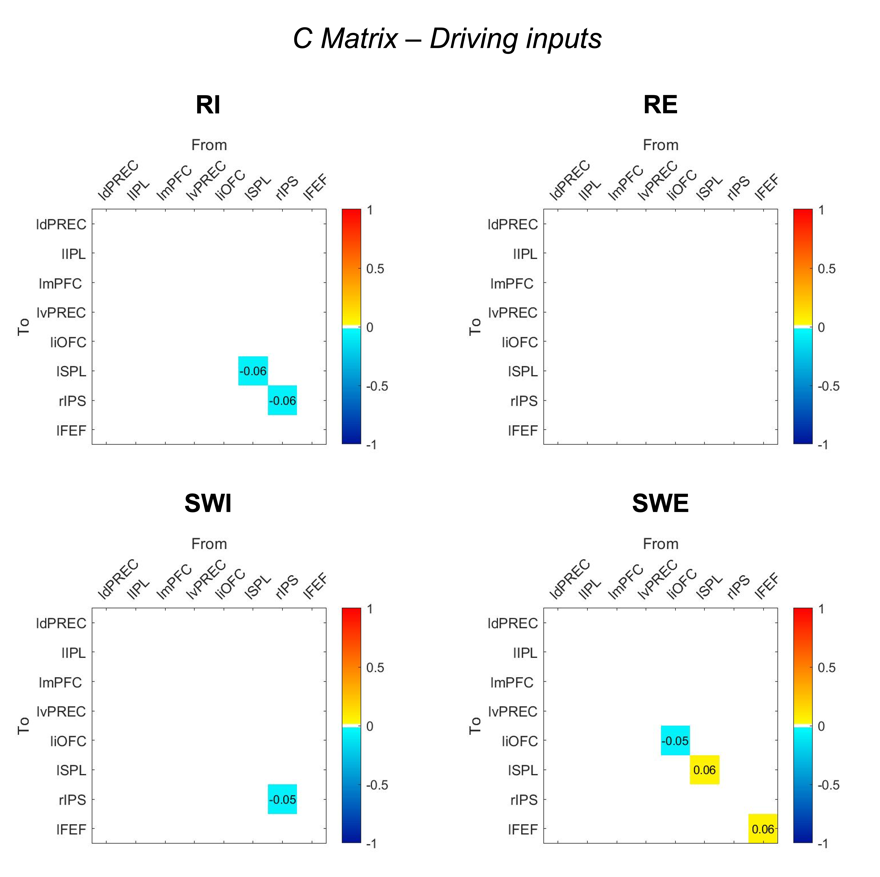


**Figure S9.** Driving inputs from the task (C matrix), representing where each task condition enters the network. We report the connections that exceeded the 95% posterior probability threshold in the PEB analysis. Warm colours indicate a positive drive from task condition while cold colours indicate a negative drive. By definition, driving inputs are only present in self-connections. Abbreviations: RI = internal repetitions; RE = external repetitions; SWI = switches to internal; SWE = switches to external; *lm*PFC = left middle prefrontal cortex; *lv*PREC = left ventral precuneus; *li*OFC = left inferior orbitofrontal cortex; *l*SPL = left superior parietal lobule; rIPS = right intraparietal sulcus; *l*FEF = left frontal eye fields; *l*IPL = left inferior parietal lobule; *ld*PREC = left dorsal precuneus.
